# A Phenome-Wide Association Study of the Effects of *Fusarium graminearum* Transcription Factors on Fusarium Graminearum Virus 1 Infection

**DOI:** 10.3389/fmicb.2021.622261

**Published:** 2021-02-11

**Authors:** Jisuk Yu, Kook-Hyung Kim

**Affiliations:** ^1^Plant Genomics and Breeding Institute, Seoul National University, Seoul, South Korea; ^2^Department of Agricultural Biotechnology, College of Agriculture and Life Sciences, Seoul, South Korea; ^3^Research Institute of Agriculture and Life Sciences, Seoul National University, Seoul, South Korea

**Keywords:** *Fusarium graminearum*, transcription factor, mycovirus, FgV1, phenome

## Abstract

The Fusarium graminearum virus 1 (FgV1) causes noticeable phenotypic changes such as reduced mycelial growth, increase pigmentation, and reduced pathogenicity in its host fungi, *Fusarium graminearum*. Previous study showed that the numerous *F. graminearum* genes including regulatory factors were differentially expressed upon FgV1 infection, however, we have limited knowledge on the effect(s) of specific transcription factor (TF) during FgV1 infection in host fungus. Using gene-deletion mutant library of 657 putative TFs in *F. graminearum*, we transferred FgV1 by hyphal anastomosis to screen transcription factors that might be associated with viral replication or symptom induction. FgV1-infected TF deletion mutants were divided into three groups according to the mycelial growth phenotype compare to the FgV1-infected wild-type strain (WT-VI). The FgV1-infected TF deletion mutants in Group 1 exhibited slow or weak mycelial growth compare to that of WT-VI on complete medium at 5 dpi. In contrast, Group 3 consists of virus-infected TF deletion mutants showing faster mycelial growth and mild symptom compared to that of WT-VI. The hyphal growth of FgV1-infected TF deletion mutants in Group 2 was not significantly different from that of WT-VI. We speculated that differences of mycelial growth among the FgV1-infected TF deletion mutant groups might be related with the level of FgV1 RNA accumulations in infected host fungi. By conducting real-time quantitative reverse transcription polymerase chain reaction, we observed close association between FgV1 RNA accumulation and phenotypic differences of FgV1-infected TF deletion mutants in each group, i.e., increased and decreased dsRNA accumulation in Group 1 and Group 3, respectively. Taken together, our analysis provides an opportunity to identify host’s regulator(s) of FgV1-triggered signaling and antiviral responses and helps to understand complex regulatory networks between FgV1 and *F. graminearum* interaction.

## Introduction

Virus divert many cellular resources to produce virus-specific components and counteract to host defense responses during virus infection (Carrera and Elena, 2012). This virus-host interaction often leads to the expression of disease symptoms in the host by triggering physiological alteration and modifying cytoskeleton or membrane structures (Osterbaan and Fuchs, 2019).

Transcription factors (TFs) are DNA-binding proteins responsible for modulating gene regulatory systems by cooperating with a range of proteins, including other upstream or downstream TFs, transcription initiation complex, and epigenetic regulators (Spitz and Furlong, 2012; Shelest, 2017; Mitsis et al., 2020). During virus-host interaction, TFs are directly or indirectly regulate defense response by activation or repression of downstream signaling pathways (Alves et al., 2014). In plant, members of TF families belonging to WRKY family, myeloblastosis related proteins (MYB), basic leucine zipper (bZIP), APETELA2/Ethylene-Responsive Factor (AP2/ERF) family, and NAC transcription factors have been shown to be associated with defense response against plant viruses as well as abiotic stress responses (Alves et al., 2014; Ng et al., 2018).

A filamentous fungus *Fusarium graminearum* causes Fusarium head blight of major cereal crops, such as wheat, barley, and rice (Dweba et al., 2017). *Fusarium* species also produce mycotoxins such as deoxynivalenol (DON), nivalenol, and zearalenone that are considered threat to the animals and human health (Ferrigo et al., 2016). Since the report of the complete genome sequence of *F. graminearum*, many researchers have attempt to characterize function(s) of TFs and their target genes in gene regulatory network using diverse computational and experimental approaches (Son et al., 2011; Liu et al., 2019; Guo et al., 2020). Systematic loss-of function studies and transcriptomic studies under comparable condition provide new insights into the role of TFs in complex regulatory networks for mycotoxin biosynthesis, sexual development, and virulence in *F. graminearum* (Son et al., 2011; Kim et al., 2015; Kazan and Gardiner, 2018; Chen Y. et al., 2019; Guo et al., 2020). Their interconnection and specific roles on signaling pathways, however, are largely unknown. In previous study, to determine the functions and interconnectedness of individual TFs, the gene-disruption library of 657 potential TF genes in *F. graminearum* was constructed and analyzed (Son et al., 2011). Each of TF deletion mutant was categorized by phenotypic characteristics, such as mycelial growth, sexual and asexual developments, virulence, toxin production, and stress responses (Son et al., 2011).

Fusarium graminearum virus 1 (FgV1) is a single-stranded RNA (ssRNA) virus and closely related to the proposed family “*Fusariviridae*” (Kwon et al., 2007; Honda et al., 2020). FgV1 infection causes remarkable phenotypic change such as reduced growth rate, increased pigmentation, reduced mycotoxin synthesis, reduced pathogenicity, and defects in sexual development in *F. graminearum* (Chu et al., 2002; Lee et al., 2014). Previous literature review provides a summary of fungal host proteins that might be associated with FgV1 accumulation, mycovirus transmission, and symptom development in *F. graminearum* (Yu and Kim, 2020). FgHex1 that functions in the maintenance of cellular integrity enhances the FgV1 RNA synthesis by binding to the FgV1 genomic RNA (Son et al., 2016b). FgHal2 is required for the vegetative growth and methionine biosynthesis of *F. graminearum* and *FgHal2* gene deletion reduces FgV1 RNA accumulation and vertical transmission of virus (Yu et al., 2015; Yun et al., 2015). Transcriptional reduction of *FgSWI6*, encoding possible transcription cofactor in *F. gramimearum*, following FgV1 infection might be related to the changes of fungal host colony morphology caused by FgV1 infection (Son et al., 2016c). However, these studies focused on biological functions of individual components, and specific biological processes and pathways remains elusive. Using transcriptomic analysis, we previously demonstrated that numerous *F. graminearum* genes including transcription factors were differentially expressed upon FgV1 infection (Lee et al., 2014). Recent study reported that FgV1 protein pORF2 could inhibit transcriptional induction of *FgDICER* and *FgAGO* genes to counteract host’s antiviral RNA silencing response (Yu et al., 2020). This previous study proposed that FgV1 might be able to affect gene regulatory networks directly or indirectly, which lead to pleiotropic phenotypes on the presence of significant amount of viral RNA in fungal host.

Here, to gain new insights on the role(s) of host transcription factors that might be associated with viral RNA replication or symptom development following FgV1 infection, we transferred FgV1 to gene-deletion mutant library of 657 putative TFs in *F. graminearum*. Based on this library, we analyzed phenotype of virus-infected mutants and the relationship between FgV1 RNA accumulation and phenotypic differences of FgV1-infected TF deletion mutants. To our knowledge, this is the first description of phenome-based association study in characterizing effects of *Fusarium graminearum* transcription factors on FgV1 infection.

## Materials and Methods

### Fungal Strains and Growth Condition

*Fusarium graminearum* GZ03639 WT strain and 657 TF deletion mutant library were provided by the Center for Fungal Genetic Resources (Seoul, South Korea). All fungal isolates were stored in 20% (v/v) glycerol at −80°C and TF deletion mutants were reactivated at 25°C on potato dextrose agar (PDA) with geneticin (50 μg/ml). TF deletion mutants were subcultured on complete medium (CM) agar containing geneticin for further experiment. Fungal colonies incubated on CM agar at 25°C for 120 h were photographed. Fungal cultures used for extraction of RNA were prepared as previously described (Lee et al., 2014). Briefly, freshly grown mycelia was inoculated into CM broth, and the cultures were incubated at 25°C for 120 h. Hyphae were collected by filtering through 3 MM paper followed by washing with distilled water, dried by blotting mycelia between paper towels, and frozen at −80°C.

### Virus Transmission

FgV1-infected *F. graminearum* GZ03639 (WT-VI) was generated by using protoplast fusion method (Lee et al., 2011). We confirmed FgV1 infection by total RNA extraction and reverse transcription polymerase chain reaction (RT-PCR) using virus specific primer pair and selected WT-VI as positive control for further experiments. FgV1 was introduced into TF deletion mutant by hyphal anastomosis between WT-VI and TF deletion mutant library. An agar block of WT-VI and individual TF deletion mutant strain was placed on CM agar media and incubated at 25°C for 4 days. Overlapped region of two fungal strains were isolated, transferred to CM agar contained geneticin (50 μg/ml), and subcultured twice to eliminate unstable virus-infected colony. Multiple replicates of all virus-infected mutant strains have obtained. After virus transmission has failed during at least three times repetition, we determined these TF deletion mutant strains as non-transmissible *via* hyphal anastomosis.

### Measurement of Mycelial Growth

For phenotype analysis, virus-infected TF deletion mutants were photographed after 5 days of cultivation (Supplementary Figure 1). Radial growth of mycelia from the inoculum was measured using ImageJ software (Schneider et al., 2012). The TF deletion mutants that showed reduced mycelial growth after gene deletion was also assessed (Supplementary Table 1).

### Preparation of Total RNA Samples and cDNA Synthesis

For nucleic acid extraction, frozen mycelia were pulverized using liquid nitrogen and a mortar and pestle. Total RNAs were extracted with RNAiso Plus reagent (Takara Bio, Shiga, Japan) followed by treatment with *DNase*I (Takara Bio) to remove genomic DNA according to the manufacturer’s instructions. As described previously, 4 M LiCl was added to total RNA extract to a final concentration of 2 M to isolate ssRNA fraction (Yu et al., 2018). Samples were then incubated at −20°C for 2 h, ssRNA pellets were washed in 75% ethanol and suspended in RNase-free water. Next, 3 μg of ssRNA of each sample was used to synthesize first-strand cDNA with an oligo (dT)_18_ primer and GoScript^TM^ reverse transcriptase (Promega, Madison, WI, United States) according to the manufacturer’s protocols. All synthesized cDNAs were diluted to 20 ng of mixture with nuclease-free water.

### Real-Time RT-PCR Analysis

Real-time quantitative RT-PCR (qRT-PCR) was performed with a Bio-Rad CFX384^TM^ Real-time PCR system using gene-specific internal primers as described previously with slight modification (Yu et al., 2018). Each reaction mix (10 μl) consisted of 20 ng of cDNA, 5 μl of 2 X iQ^TM^SYBR^®^ Green Supermix (Bio-Rad, Hercules, CA, United States), and 10 pmoles of each primer. The thermal profile was as follows: 3 min at 95°C and 40 cycles of 10 s at 95°C, 30 s at 59°C, and melting curve data obtained by increasing the temperature from 55 to 95°C. Two endogenous reference genes, i.e., ubiquitin C-terminal hydrolase (*UBH*, locus FGSG_01231) and elongation factor 1α (*EF1*α, locus FGSG_08811), were used as internal controls to normalize qRT-PCR results. Data were analyzed using the Bio-Rad CFX Manager V1.6.541.1028 software (Bio-Rad). RNA samples were extracted from at least two independent, biologically replicated experiments, and each PCR product was evaluated in at least three independent experiments, including three technical replicates. All primer sets used in this study are listed in Supplementary Table 2.

### Viral dsRNA Confirmation and Semi-Quantification

Three micrograms of *DNase*I-treated total RNAs from all virus-infected TF deletion mutants were treated by 30 units of S1 Nuclease (Takara Bio). Samples were loaded into 1% agarose gel for analysis of viral double-stranded (dsRNA) accumulation. After separation on agarose gel, dsRNA was visualized in a UV transilluminator. To measure relative accumulation of FgV1 viral dsRNA in TF deletion mutants, 3 μg of total RNA from all virus-infected mutants were loaded and separated on 1% agarose gel. Ethidium bromide-stained gels were visualized in a UV transilluminator. Band intensity were measured using ImageJ software (Schneider et al., 2012). The relative amount of viral genomic dsRNA was estimated by measuring the amount of FgV1 RNA relative to 18S rRNA.

## Results

### Phenotype Analysis of FgV1-Infected TF Gene-Deletion Mutants

To investigate the effect of TF genes on FgV1 infection in *F. graminearum*, we transferred FgV1 to putative 657 TF gene deletion mutants. Among 709 TF genes, 657 TF genes were successfully disrupted and other 52 TF genes were excluded due to lethality or technical problem of generation of homologous recombination construct (Son et al., 2011). FgV1 could effectively transmitted by hyphal anastomosis between FgV1-infected strain GZ03639 (WT-VI) strain and virus-free TF deletion mutant strains. Among total of 657 TF deletion mutants, we could not transmit FgV1 onto a 17 TF deletion mutants despite repeated trials (Table 1 and Supplementary Table 1). Representative image of colony morphologies for each FgV1-infected TF deletion mutants (total 640) were shown in Supplementary Figure 1. Typically, colony morphology of WT-VI (a FgV1-infected strain) includes irregular colony shape, no aerial mycelium, and dense mycelia with deep red or brown color. Most of FgV1-infected TF deletion mutants showed similar colony morphologies compared to that of WT-VI, but several virus infected fungal colonies showed abnormal colony morphology (e.g., slower or faster mycelial growth, low density and scarce hyphal growth, curly mycelia, aerial mycelia development, and change of pigment production).

**TABLE 1:** Analysis of FgV1-infected transcription factor deletion mutants.

TF Classification	TF	ΔTF	TF-FgV1	Group^a^		
				
				1	2	3
bHLH	16	15	15	0	11	4
bZIP	22	22	22	0	21	1
C2H2 zinc finger	98	94	94	5	84	5
Heteromeric CCAAT factors	8	8	8	3	5	0
HMG	37	34	34	3	30	1
Homeodomain-like	14	7	7	1	6	0
Nucleic acid-binding, OB-fold	47	40	40	4	35	1
Winged helix repressor DNA-binding	27	26	26	0	23	3
Helix-turn-helix, AraC type	8	7	7	0	6	1
GATA type zinc finger	8	7	7	0	7	0
Zinc finger, CCHC-type	12	12	12	0	12	0
Zn2Cys6 zinc finger	316	296	286**^b^**	16	254	16
Myb	19	17	17	1	13	3
Others	77	72	65**^b^**	2	52	11
Total	709	657	640	35	559	46

*^a^Groups 1–3 were determined by mycelial growth on complete media (CM). Average radial growth on CM of FgV1-infected TF deletion mutants compared to virus-free strain or wild-type (WT) strain (set to a value of 100) was divided as groups (Group 1, less than 33; Group 2, 33–62.9; Group 3, 63–100 of WT strain. ^b^Some of TF deletion mutants did not obtain FgV1 through hyphal anastomosis.*

**FIGURE 1:**
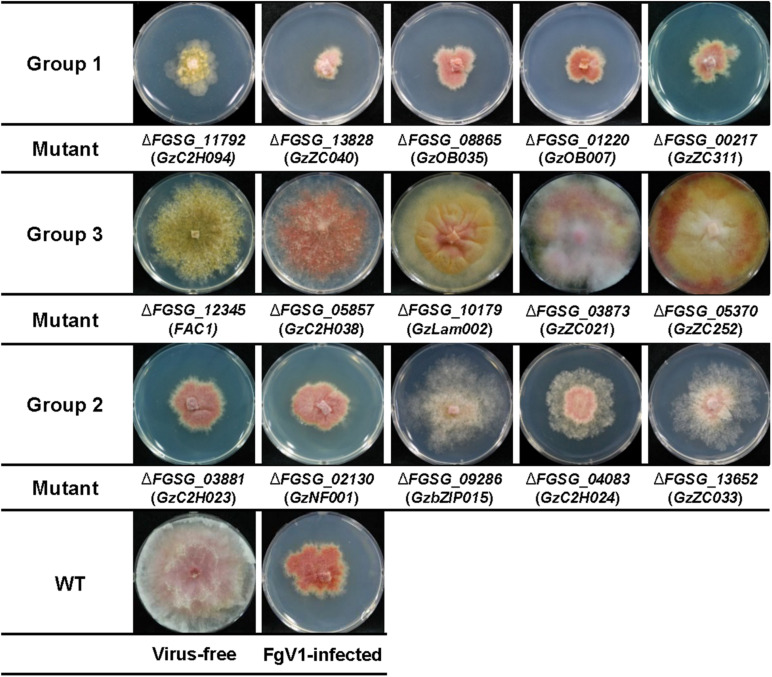
Representative FgV1-infected colony morphology according to mycelial growth. Representative examples of colony morphology of FgV1-infected TF deletion mutants are shown. All cultures were photographed after incubating 5 days on complete media (CM). WT, *Fusarium graminearum* wild-type (WT) strain GZ03639. The virus-free TF deletion mutants that correspond to FgV1-infected TF deletion mutants in this figure did not changed mycelial growth compared to virus-free WT.

FgV1-infected TF deletion mutants were classified into three groups according to the mycelial growth phenotype compared to that of WT-VI or virus-free TF deletion mutant (Figure 1 and Table 1). For this, the mycelial length of WT-VF was set to a value of 100 (±11). Mycelial length of WT-VI decreased to 40–55 (average 47.5 ± 7) compared to that of WT-VF. In case of TF deletion mutant showing growth retardation, this virus-free TF deletion mutant was used as standard to compare FgV1-infected TF mutant. Comparing with each virus-free TF deletion strain or wild-type (WT) strain, we classified Group 1 and 3 of FgV1-infected TF deletion mutants determined by growth under 33 (0–32.9) or over 63 (63–100), respectively. The FgV1-infected TF deletion mutants in Group 1 indicated slow or weak mycelial growth compared to that of WT-VI. Among FgV1-infected TF deletion mutants in Group 2 (mycelial growth, 33–62.9), we could divide them into two subgroups. One showed similar colony morphology of WT-VI while the other subgroup did not follow typical phenotype WT-VI and showed fluffy but low density of mycelia phenotype. In contrast, Group 3 consists of virus-infected TF deletion mutants showing mild symptoms, such as faster mycelial growth and partially restored aerial mycelia formation.

Approximately 88% of FgV1-infected TF deletion mutants (Group 2) showed similar phenotype and mycelial growth regardless of phenotype of virus-free TF gene deletion mutants. Among FgV1-infected TF deletion mutants in Group 3, which growth reduction rate was lower than 10%, colony morphology of most mutants in this group showed recovery phenotype and viral accumulation level was as low as 10% of WT-VI although FgV1 infection might still affect colony morphology. In this regard, comparisons of colony morphology including mycelial growth of TF deletion mutants following hypovirulence-associated FgV1 infection help screening host gene(s) that might attribute or affect development of virus-derived symptom and FgV1 replication. We observed that *F. graminearum* TF candidates belong to the groups of bHLH (basic–helix–loop–helix) motif and heteromeric CCAAT-binding factor showed relatively high phenotypic variation following FgV1 infection compared to that of other TF families (Table 1).

### TF Factors That Might Be Involved in FgV1-Derived Host Symptom

**FIGURE 2:**
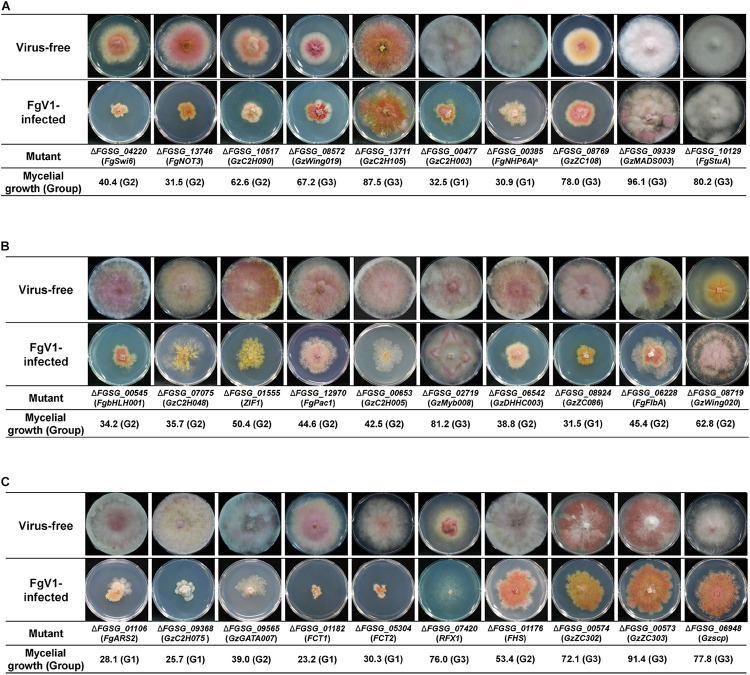
Selected colony morphology of FgV1-infected TF deletion mutants. Colony morphology of virus-free and of FgV1-infected TF gene-deletion mutant strains. **(A)** TF gene-deletion mutants that showed multiple defect phenotypes after single gene deletion. **(B)** TF gene-deletion mutants that related to sensitive response against abiotic stress factor. **(C)** TF gene-deletion mutants that related to DNA damage response. All cultures were photographed after 5-day incubation on CM.

Previous study analyzed phenotypes of putative 657 TF deletion mutants and divided them based upon their major phenotypic categories such as mycelial growth, sexual development, conidia production, toxin production, and stress responses (Son et al., 2011). This phenome-based analysis demonstrated that fungal virulence, growth, and DON production were highly correlated with sexual development (Son et al., 2011). Because FgV1 infection causes multiple phenotypic alterations, this simultaneous and multiple FgV1-derived symptom might also be consequences of interaction between virus and host factor that have pivotal roles in gene regulatory network. In this regard, we selected 35 TF deletion mutants that exhibit multiple defects in mycelial growth, virulence, sexual development, and toxin production (Figure 2 and Supplementary Table 3; Son et al., 2011). Ten out of 35 selected TF deletion mutants that were not related with environmental stress responses or DNA damages were shown in Figure 2A, except *FgNHP6A* (FGSG_00385) deletion mutant that showed pH 4-resistance. Among these 35 TF deletion mutants, most of gene deletion mutants showed decreased mycelial growth compared to WT. Colony morphology of virus-free deletion mutants including *FgSWI6* (FGSG_04220), *FgNOT3* (FGSG_13746), *GzC2H090* (FGSG_10517), *GzWing019* (FGSG_08572), and *FgCrz1A* (FGSG_13711) showed similar phenotypes to those of WT-VI such as reduced aerial mycelia, reduced mycelial growth, and increased pigmentation (Figure 2A and Supplementary Table 1, compare to WT-VI in Figure 1). When we confirmed gene expression level of these five TFs after FgV1 infection by qRT-PCR, those of all five TFs showed decreased level compared to WT-VF (Supplementary Table 4). Comparing phenotype changes upon FgV1 infection in Group 1, *GzC2H003* (FGSG_00477) and *FgNHP6A* (FGSG_00385) TF deletion mutants showed slow mycelial growth after FgV1 infection compared to WT-VI (Figure 2A, compare to WT-VI in Figure 1). On the contrary, mycelial growth in virus-infected TF deletion mutants in Group 3, *FgCrz1A* (FGSG_13711), *GzZC108* (FGSG_08769), *GzMADS003* (FGSG_09339), and *FgStuA* (FGSG_10129) showed little reduction of mycelial growth compared to WT-VI. Six out of those 35 TF deletion mutants including *GzAPSES004* (FGSG_10384) and *FgFSR1* (FGSG_01665) mutants were not able to uptake FgV1 *via* hyphal anastomosis method, this result might be explained as growth defect in those deletion mutants (Supplementary Figure 1).

### Comparison of Phenotypes Produced in Response to Deletion and FgV1 Infection of the TFs Associated With Environmental Stress Response

TF phenome analysis described phenotypic change under diverse abiotic stresses, including osmotic stress, reactive oxygen species (ROS) stress, fungicide, cell wall stress, and acidic (pH = 4) or basic (pH = 11) conditions (Son et al., 2011). We compared TF phenotypes in response to those stress factors, considering FgV1 infection as biotic stress in *F. graminearum*, whether TF gene disruptions that showed different response to abiotic stress might also relate to response against to the FgV1 infection (Figure 2B). In results, among pH 11-sensitive or pH 11-resistant TF deletion mutants, *GzbHLH001* (FGSG_00545), *GzC2H048* (FGSG_07075), and *ZIF1* (FGSG_01555) deletion mutants showed more damaged virus-infected phenotype compared to WT-VI though they were belong to the Group 2. The other pH 11 responsive gene *FgPac1* (FGSG_12970) deletion mutant showed similar phenotype to WT-VI. Δ*GzC2H048* and Δ*ZIF1* also showed osmotic stress response. Between two pH 4-resistance TF deletion mutants, Δ*GzC2H005* (FGSG_00653) and Δ*GzMyb008* (FGSG_02719), they showed different phenotype. FgV1-infected Δ*GzC2H005* deletion mutant showed very weak and low density of colony morphology. In contrast, FgV1-infected Δ*GzMyb008* (Group 3) showed much faster mycelial growth and formed rhombus-shape of red line at the outside region of colony. In case of *GzDHHC003* (FGSG_06542) and *GzZC086* (FGSG_8924), related with oxidative stress response, showed normal growth but with relatively reduced mycelia growth compared to WT-VI. *FgFlbA* (FGSG_06228) showed increased transcript level following FgV1 infection and deletion mutant showed resistance phenotype in all stress factors except for pH 11 stress response. The virus-infected Δ*FgFlbA* (Group 2) colony grow normally but contain clustering region around fungal colony. In contrast, while Δ*GzWing020* (FGSG_08719, Group 3) showed sensitive responses to all stress responsive factors in phenome data, FgV1-infected colony showed increased aerial mycelia production and hyphal growth compared to WT-VI. Obtained results indicated that some of *F. graminearum* TF candidates that showed sensitive response in pH, fungicide or ROS stress alone also involved in response to FgV1 infection as well as TF candidates that response in broad range of environmental stress factors.

### The Relationship Between FgV1 Infection and TFs Involved in DNA Damage Response

Previous study identified 16 putative TFs involved in DNA damage responses (DDRs) (Son et al., 2016a). In this study, we found that these DDR TF gene deletion group included relatively high portion of FgV1-infected TF deletion mutants that belong to Groups 1 and 3. Among 13 FgV1-infected TF, four FgV1-infected TF deletion mutants including *FgARS2* (FGSG_01106), *GzC2H075* (FGSG_09368), *FCT1* (FGSG_01182), and *FCT2* (FGSG_05304) were divided in Group 1. FgV1-infected Δ*GzGATA007* (FGSG_09565) belong to Group 2, however, showed retarded growth compared to WT-VI or to the typical colony morphologies of FgV1-infected TF deletion mutants belonging to Group 2 (Figure 2C, compare to WT-VI in Figure 1). *GzC2H075* (FGSG_09368) and *GzGATA007* (FGSG_09565) deletion mutants, which showed apparent reduction in mycelial growth following FgV1 infection, exhibited sensitive response only to DNA damage reaction in phenome data. Individual mycelial growth value of *RFX1* (FGSG_07420; Group 3) deletion mutant was slightly higher than that of WT-VI, however, virus-infected mutant showed strong inhibition of mycelial growth phenotype so we are unable to process further experiment. *FHS* (FGSG_01176; Group 2) deletion mutant that showed oxidative stress and ROS response along with DDR did not show significant change of colony morphology following FgV1 infection. The colony morphology of *GzZC302* (FGSG_00574; Group 3) and *GzZC303* (FGSG_00573; Group 3) deletion mutants showed similar reduced aerial mycelia, increased pigmentation, and responded to multiple stress factors include oxidative, ROS and pH, however, their virus-infected phenotypes were not significantly different compared to WT-VI. Δ*Gzscp* (FGSG_06948; Group 3), which exhibit multiple defects along with DDR, showed little reduction of mycelial growth compared to WT-VI.

Although all DDR-related putative TF genes exhibited different sensitivity to DNA damaging agent include methyl methanesulfonate, hydroxyurea, bleomycin, and camptothecin (Son et al., 2016a), we could not correlate a common DNA damaging agent that links to displayed phenotype among FgV1-infected TF deletion mutants belong to Group 1.

### Comparisons With RNA-Seq Data and Phenome Data

Previous study demonstrated that 24 TF genes were up- or down-regulated following FgV1 infection using transcriptomics-based analysis (Lee et al., 2014). We validated these RNA-Seq data with selected TF genes in this study (Supplementary Table 5). Among those 24 TF genes, only two TF genes including *GzZC252* (FGSG_05370) and *GzZC311* (FGSG_00217) were grouped into 1 and 3, respectively, following FgV1 infection (Figure 1). Interestingly, expression of both *GzZC252* (FGSG_05370) and *GzZC311* (FGSG_00217) genes were up-regulated upon FgV1 infection (Lee et al., 2014; Supplementary Table 5). Δ*GzZC311* did not show significant phenotypic change in mycelial growth compared to WT. Phenotype of FgV1-infected Δ*GzZC311* showed slow growth of mycelia compared to that of WT-VI. In contrast, Δ*GzZC252-*VF showed flat colony morphology with scarce growth of aerial mycelia but regular growth of mycelial growth in length. FgV1-infected Δ*GzZC252* did not decrease mycelial growth but caused color change from pale yellow to dark yellow in overall area of culture plate. The other 21 TF gene deletion mutants showed similar colony morphology compared to WT-VI. Although RNA-Seq analysis also identified *GzbHLH006* (FGSG_02516), *GzbHLH007* (FGSG_02814) *GzC2H006* (FGSG_00764), *TRI15* (FGSG_03881), and *GzGATA003* (FGSG_04626) that showed significant changes of gene expression levels upon FgV1, Fusarium graminearum virus 2 (FgV2; a Chrysovirus), FgV3 (a Fusagravirus), and Fusarium graminearum hypovirus 1 infections (Lee et al., 2014; Wang et al., 2016), those deletion mutants did not show significant change of colony morphology following FgV1 infection. In addition, we selected several TF genes in Groups 1 and 3 for confirmation of change of gene expressions following FgV1 infection (Supplementary Table 4). In Group 1, *FGSG_08865* and *FGSG_13828* genes showed significantly increased expression levels following FgV1 infection among 5 genes. In Group 3, expression levels of *FGSG_09339, FGSG_08455*, and *FGSG_03873* genes were decreased while expression level of FGSG_12742 was increased compared to that of WT-VI. These results showed FgV1 infection affects the expression levels of some putative TF genes, however, all of these changes might not be directly related with FgV1 accumulation or FgV1-mediated colony morphology in *F. graminearum*.

### TFs That Might Be Involved in FgV1 RNA Accumulation

**FIGURE 3:**
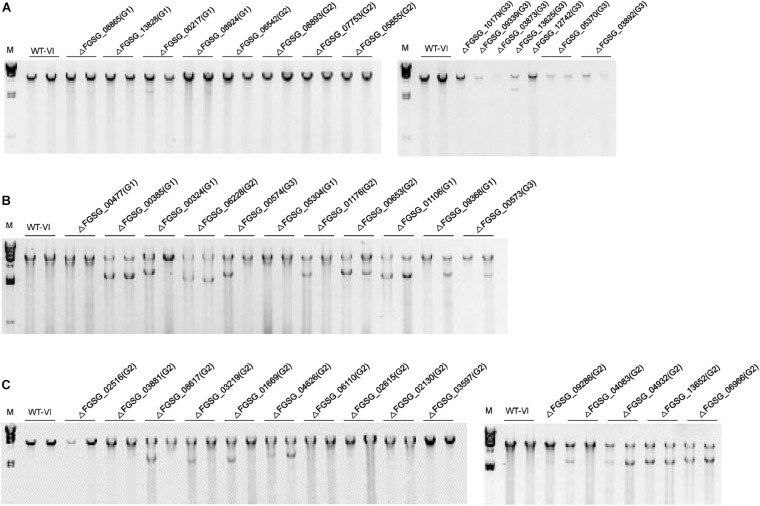
Accumulation of FgV1 viral double-stranded RNA in virus-infected TF deletion mutant strains of *F. graminearum*. **(A)** dsRNA accumulation of TF gene-deletion mutants that belong to Groups 1 to 3. **(B)** TF gene-deletion mutants that showed multiple defect phenotypes after single gene deletion, TF gene-deletion mutants related to sensitive response against abiotic stress factor and DNA damage response. **(C)** TF gene-deletion mutants showing significant changes of gene expression levels upon FgV1 (left) and TF gene-deletion mutants showing abnormal colony morphology in Group 2 (right). Number in parenthesis represents group of each sample. A 3 μg quantity of total RNAs per sample was treated with *DNase*I and S1 Nuclease and separated in 1% agarose gel. The largest band in the each sample represents the full-length FgV1 dsRNA (6.6 kb); smaller bands indicate internally deleted forms of viral dsRNA. The lane marked M correspond to lambda DNA digested with *Hin*dIII. WT-VI, FgV1-infected WT GZ03639 strain.

**FIGURE 4:**
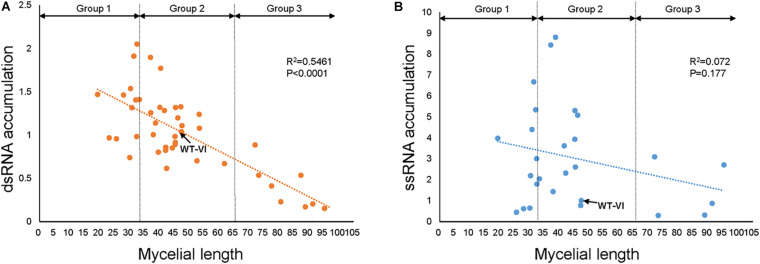
Relationship between mycelial growth and the viral RNA accumulation in TF deletion mutants. Relationship between radial growth of mycelia and FgV1 RNA replication was estimated by calculating the viral dsRNA **(A)** and ssRNA **(B)** accumulations in FgV1-infected TF deletion mutants. Dotted and dashed lines indicate the linear regression between the mycelial growth and viral RNA accumulation.

To isolate TFs that might be associated with viral replication, we confirmed dsRNA and viral ssRNA accumulation levels in FgV1-infected TF deletion mutants (Table 2). We selected several FgV1-infected TF deletion mutants belong to Groups 1, 2, and 3. Selected isolates include FgV1-infected mutants with phenotypic changes such as defect in sexual development, TF genes responsive to stress or DNA damage, and significantly up- or down-regulated TF genes upon FgV1 infection from RNA-Seq analysis. In results, increased viral ssRNA accumulation level was observed in *FgNHP6A* (FGSG_00385), *GzZC040* (FGSG_13828), *GzZC086* (FGSG_8924), *TRI15* (FGSG_03881), *FgFlbB* (FGSG_03597), *GzDHHC003* (FGSG_06542), and *GzZC267* (FGSG_01669) deletion mutants. Among them, dsRNA accumulation level was also significantly increased in FgV1-infected *GzZC086* deletion mutants. Δ*GzZC021* (FGSG_03873), *GzZC252* (FGSG_05370), and Δ*GzZC303* (FGSG_00573) in Group 3 showed significant decrease in viral dsRNA accumulation level. FgV1-infected Δ*GzZC252* showed significant reduction in dsRNA accumulation but not in viral ssRNA accumulation level compared to that of WT-VI. In addition, we observed dsRNA patterns of these TF deletion mutants (Figure 3). TF deletion mutants classified into Group 3 including Δ*GzZC021* (FGSG_03873), Δ*GzZC252* (FGSG_05370), and Δ*GzZC197* (FGSG_03892) showed decreased dsRNA accumulation compared to those of WT-VI and other mutants classified into Groups 1 and 2 (Figure 3A). Defective RNAs (D-RNAs, approximately 2–3 kbp long) were often observed in TF gene deletion mutants that showed multiple phenotypic changes and related with stress or DNA damage responses (Figure 3B). Among TF deletion mutants in Group 2, FgV1-infected mutants including Δ*GzbZIP015* (FGSG_09286), Δ*GzC2H024* (FGSG_04083), and Δ*GzZC033* (FGSG_13652) produced fluffy but low density of aerial mycelia (Figure 1) and also accumulated D-RNAs during FgV1 replication (Figure 3C, right panel). These results indicated that deletion of single TF gene affects FgV1 replication at different step(s) and generation of D-RNAs. In Figure 1, we simplify FgV1-infected TF deletion mutants by grouping based upon mycelial growth rate as the first step. We postulated that mycelial growth and viral RNA accumulation might inversely correlated in FgV1-infected fungal strains if mycelial growth of mutant did not changed by target gene deletion. To examine relationship between mycelial growth and viral RNA accumulation, we plot dsRNA or ssRNA accumulation (y) against mycelial length (x) using selected FgV1-infected TF deletion mutants (Figure 4). In general, FgV1-infected TF deletion mutants that grew slower than WT-VI accumulated higher level of viral dsRNA compared to WT-VI. In contrast, FgV1-infected TF deletion mutants that grow faster than WT-VI accumulated lower level of viral dsRNA compared to WT-VI. Altogether, this result indicates that the relative levels of viral dsRNA accumulation in fungal colonies negatively correlate with mycelial growth of FgV1-infected TF mutant.

**TABLE 2:** Comparisons of relative ratio of mycelial growth, dsRNA accumulation, and ssRNA accumulation of FgV1-infected TF deletion mutants.

	Mycelial length^a^	dsRNA^b^	ssRNA^c^	Note^d^
**WT-VI**	47.5	1.04 ± 0.1	0.98 ± 0.1	
**Group 1**				
Δ*FGSG_00477*	32.5	0.98 ± 0.2	3.01 ± 0.9	MD
Δ*FGSG_01106*	28.1	1.46 ± 0.7	0.62 ± 0.02	MD, DDR
Δ*FGSG_09368*	25.7	0.96 ± 0.2	0.45 ± 0.1	DDR
Δ*FGSG_00385*	30.9	1.32 ± 0.1	4.41 ± 1.4*	MD, pH4(R)
Δ*FGSG_08865*	30.4	1.54 ± 0.3	2.19 ± 0.7	N
Δ*FGSG_13828*	19.4	1.47 ± 0.8	3.98 ± 1.8*	N
Δ*FGSG_08924*	31.5	1.91 ± 0.6*	6.68 ± 3.0*	Fung, virus response
Δ*FGSG_00217*	32.2	1.41 ± 0.4	5.34 ± 2.5*	Virus response
Δ*FGSG_00324*	32.6	2.05 ± 1.3	1.79 ± 1.0	MD, os
**Group 2**				
Δ*FGSG_09286*	46.3	1.20 ± 0.2	5.09 ± 2.4	Virus response
Δ*FGSG_03881*	41.7	1.28 ± 0.3	3.62 ± 1.7*	Virus response
Δ*FGSG_04083*	47.3	1.33 ± 0.1	0.78 ± 0.2	Virus response
ΔFGSG_08617	42.2	0.86 ± 0.3	2.32 ± 0.4	Virus response
Δ*FGSG_08893*	37.2	1.26 ± 0.1	8.44 ± 0.7*	N
Δ*FGSG_06110*	45.5	1.32 ± 0.01	2.61 ± 0.6*	Virus response
ΔFGSG_02615	33.4	1.41 ± 0.2	2.05 ± 0.5	Virus response
Δ*FGSG_03597*	45.3	0.98 ± 0.1	3.94 ± 1.1*	N
Δ*FGSG_06542*	38.8	1.14 ± 0.5	8.81 ± 5.2*	MD, ROS
Δ*FGSG_11686*	38.0	1.00 ± 0.1	1.43 ± 0.2	N
Δ*FGSG_01669*	45.3	0.89 ± 0.3	5.31 ± 0.5*	Virus response
**Group 3**				
Δ*FGSG_03873*	88.9	0.17 ± 0.2**	0.31 ± 0.1**	N
Δ*FGSG_13625*	73.3	0.54 ± 0.2	0.29 ± 0.1**	N
Δ*FGSG_05370*	95.3	0.15 ± 0.1**	2.71 ± 0.2*	Virus response
Δ*FGSG_00574*	72.1	0.89 ± 0.1	3.10 ± 0.5*	MD, DDR, Os,ROS,Fung,pH11
Δ*FGSG_00573*	91.4	0.20 ± 0.1**	0.87 ± 0.3	DDR, Os,ROS,Fung,pH11

*^*a*^Radial growth was measured after a 5-day incubation on CM. Average radial growth on CM of FgV1-infected TF deletion mutants compared to virus-free strain or WT strain (set to a value of 100) was divided as groups (Group 1, less than 33; Group 2, 33–62.9; Group 3, 63–100 of WT strain). ^*b*^Relative accumulation of FgV1 viral dsRNA in TF deletion mutants was measured using 3 μg total RNA samples at 120 h post-inoculation (hpi). Values were normalized to the FgV1 RNA in WT-VI (set to a value of 1), with the standard deviation based on at least two independent RNA preparations. Different number of asterisks indicate data are significantly differed (*P* < 0.05) from each other and mean for FgV1-infected GZ03639 WT strain based on LSD test. ^c^Quantification of FgV1 viral ssRNA at 120 hpi using real-time RT-PCR. cDNAs were generated from ssRNA samples obtained after 120 h of incubation. EF1α and UBH gene transcripts were used as internal controls. Values are means (+ SD) of two biological replicates with at least two experimental replication. Different number of asterisks indicate data are significantly differed (P < 0.05) from each other and mean for FgV1-infected GZ03639 WT strain based on LSD test. ^d^MD, multiple defects after TF gene deletion; DDR, TF deletion mutant related with DNA damage responses; os, sensitive in osmotic stress; ROS, sensitive in ROS stress; Fung, sensitive in fungicide response; pH4(R), resistance response against pH 4 stress; N, TF deletion mutant not showing any significant change; virus response, level of gene expression was changed after virus infection.*

## Discussion

Identifying host factors involved in FgV1-derived symptom induction and viral RNA accumulation is a key aspect of understanding the molecular mechanism during *F. graminearum*-FgV1 interactions. Previous studies suggested that viral components interfere with host cell signaling pathways and progressively cause alteration in physiological and developmental processes, which culminate in visible virus-induced symptoms (Urbanowski et al., 2008; Pesti et al., 2019). In this respect, we used genome-wide TF deletion mutant library for *F. graminearum* to find host transcription factors and host-cell signaling pathways that might be associated with pleiotropic effects of FgV1 infection on fungal host and to identify novel host factor which might be involved in FgV1 RNA accumulation in host cell.

We observed different phenotype change in fungal colony color which turns yellow after virus infection without greater reduction of mycelial growth in some TF deletion mutants. Those genes were not listed in phenome data as pH sensitive responsive mutants that showed reduced mycelial growth at pH 4 or pH 11 (Son et al., 2011). However, it is worth noting that pH also impacts on pigment production and mycelium color. For example, the red pigment of *F. graminearum* is pH sensitive and changes color from red to yellow as the pH drop (Leslie and Summerell, 2008). Because pH affects wide range of fungal physiological processes and gene expression in fungal cells (Bousset et al., 2019), change of colony morphology of those TFs deletion mutants might be related with pH stability following virus infection. In addition, dsRNA accumulation was decreased in Δ*GzZC197* (FGSG_03892) as shown in Figure 3, pH stability might also affect replication of viral RNA. Since whether pH impacts virus-host interactions is not clear in *F. graminearum*, further studies are needed.

In TF deletion mutant library, we were interested in TF deletion mutants showing pleiotropic phenotype similar with FgV1-infection derived phenotype in *F. graminearum*. We expected it would provide information for identifying host factor(s) or characterizing signaling pathway that related with hypovirulence-associated traits of FgV1 regardless of phenotype observation of FgV1-infected TF deletion mutant. As mentioned above, several TF deletion mutants showed WT-VI like colony morphology. Some of these TFs might play central roles in reprogramming transcriptional network or function as an important downstream regulator, which results in pleiotropic effects by gene deletion. For example, *FgSWI6* and *GzAPSES004* were suggested as hub regulators of virulence, mycotoxin synthesis, and sexual reproduction-associated networks (Guo et al., 2020). Previous study reported that decreased expression level of *FgSWI6* following FgV1 infection seems to be related with FgV1-derived phenotypic alteration (Son et al., 2016c). Because constitutive overexpression *FgSWI6* moderately attenuate symptom expression by FgV1 infection although it contained increased FgV1 accumulation level compared to WT (Son et al., 2016c). *FgCrz1A* is reported as a possible ortholog of *Saccharomyces cerevisiae Crz1* that has crucial role in regulating calcineurin- and Ca^2+^/calmodulin-dependent signaling (Chen L. et al., 2019). Δ*FgCrz1A* displayed multiple abnormalities in phenotypes including increased sensitivity to metal cations Ca^2+^, Mg^2+^, Mn^2+^, and Li^+^ (Chen L. et al., 2019). We observed small decrease in mycelial growth in FgV1-infected Δ*FgCrz1A* compared to WT-VI. However, including *FgCrz1A*, we are not sure whether these TF function in facilitating FgV1 replication or in regulating defense pathways of fungal host. The attempts to establish relationships between FgV1 and phenotype-associated cellular signaling pathways are required in further study.

Recent study demonstrated that *FgARS2* physically interacts with the cap-binding complex to form a stable tertiary complex (Bui et al., 2019), however, key components and regulation processes of DDR in *F. graminearum* are largely unknown. In this study, we found FgV1 infection significantly impacts on mycelial growth of several DDR-related TF deletion mutants, i.e., Δ*FgARS2* (FGSG_01106; Bui et al., 2019), Δ*FCT1* (FGSG_01182; Kim et al., 2020), Δ*FCT2* (FGSG_05304; Kim et al., 2020), Δ*GzC2H075* (FGSG_09368), Δ*GzZC303* (FGSG_00573), and Δ*GzZC302* (FGSG_00574; Figure 2). However, ss and dsRNA accumulations of those mutants were similar or slightly decreased compared to WT-VI (Table 2). These results indicated DNA damage might be induced by FgV1 infection and those DDR-related gene deletion caused significant change in mycelial growth even they contain relatively low or similar amount of viral RNA compared to WT-VI. Accordingly, genetic instability plays a considerable role in pathogenicity of FgV1 and is likely to be a key factor of FgV1-associated symptom development. In addition, we often found FgV1 infection resulted in significant accumulation of DI RNAs as were in case of Δ*FgARS2*, Δ*GzC2H075*, Δ*GzZC303*, and Δ*GzZC302* mutants. It might suggest possible role of DDR in supporting virus replication. Like other DNA viruses, some RNA viruses also have ability to trigger DDR signaling to assist host cellular conditions that are beneficial for viral replication (Ryan et al., 2016). Although it has not been determined whether FgV1 replication and symptom development are closely related with DDR in present study, this phenotype-based analysis would lead to investigate the interactions between FgV1 and the DDR in *F. graminearum* in further.

Previous transcriptome study described that 24 TF genes were differentially expressed upon FgV1 infection, however, 114 of 709 TF genes were not detected (Lee et al., 2014). Because expression levels of many TF genes vary through different phase of fungal development and environmental condition, it might has limitation in finding crucial TF genes that play crucial role(s) during FgV1 infection from transcriptome profiles obtained at a particular time-point measurement under a certain condition. Several studies analyzed transcription profiles of *F. graminearum* in response to different mycovirus infections (Lee et al., 2014; Wang et al., 2016; Bormann et al., 2018). In addition, other transcription profiles has revealed subsets of transcriptionally regulated genes using mutant strains that involved in mycotoxin synthesis, asexual or sexual development, RNAi process, abiotic stress response, and post-translational modification (Brauer et al., 2020). For examples, *GzZC196* (FGSG_03912; Group 1) and *GzZC197* (FGSG_03892; Group 3) showed reduced gene expression level in *FgDICER*s or *FgAGO*s double knockout mutant strains compared to WT (Son et al., 2017; Supplementary Table 1). *GzbHLH011* (FGSG_06262) and *GzHOMEL018* (FGSG_07243) showed reduced gene expression level in both Δ*FgGCN5* and Δ*FgSAS3* that are putative histone acetyltransferase (HATs) in *F. graminearum* (Kong et al., 2018). Both Δ*GzbHLH011* and Δ*GzHOMEL018* belong to Group 3 (Supplementary Table 1). In case of *GzZC086* (FGSG_08924), involved in oxidative stress in *F. graminearum* (Lee et al., 2018), FgV1-infected Δ*GzZC086* (Group 1) showed significantly increased viral RNA accumulation level (Table 2). Therefore, combined phenome data from this study and transcriptome data obtained from diverse conditions will help in understanding common and unique roles of TFs and signaling pathways that might be associated with host response against virus infection.

In this study, among FgV1-infected TF deletion mutants that belonged to Groups 1 and 3, we found overlapped result with transcriptome analysis. *GzZC311* (FGSG_00217) and *GzZC252* (FGSG_05370) that were grouped into 1 and 3, respectively, showed increased gene expression level following FgV1 infection but result of FgV1 transmission into each gene deletion mutant showed different colony morphology. *GzZC311* and *GzZC252* encoded hypothetical protein contain fungal-specific regulatory protein domain, however, their cellular functions have not been identified yet. Δ*GzZC252* negatively affects FgV1 dsRNA accumulation but not for ssRNA accumulation. Further investigation with complementation or overexpression mutant is required to confirm whether this gene is required for FgV1 RNA accumulation. Among Group 2, FgV1 infections in Δ*GzbZIP015* (FGSG_09286) and Δ*GzZC050* (FGSG_12597) mutants showed similar colony morphology like WT-VI (Figure 1). *GzbZIP015* encoded protein which has similarity with cross-pathway control protein 1, the ortholog of GCN4 in the yeast *S. cerevisiae*, is a main regulator of protein synthesis and might have role in longevity and stress response in *Neurospora crassa* (Hinnebusch, 2005). Given that increased expression of *GzbZIP015* gene upon FgV1 infection (Lee et al., 2014) and increased accumulation of FgV1 ssRNA in FgV1-infected Δ*GzbZIP015* mutant, *GzbZIP015* might serve as an antiviral host factor following virus infection (Table 2).

We confirmed ss and dsRNA accumulation using several TF genes deletion mutants belong to Group 2 but it showed differential gene expression upon FgV1 infection (Table 2). FgV1 ssRNA accumulation levels were increased compared to WT-VI, however, gene deletion did not seem to directly affect dsRNA accumulation. This result suggested that dsRNA accumulation level could determine FgV1-derived symptom rather than ssRNA accumulation (Figure 4).

Viruses need host factors not only to assist their replication but also to face the host antiviral defense response. Mycovirus infection in host cell boosts host antiviral response such as RNA interference (RNAi). Interestingly, Cryphonectria hypovirus 1 (CHV1) and FgV1 exhibit suppression activity against host antiviral response through suppression of RNAi component-related gene transcription (Sun et al., 2009; Yu et al., 2020). Previous studies demonstrated that the Spt–Ada–Gcn5 acetyltransferase (SAGA) transcriptional activator regulates the induction of the essential antiviral RNA-silencing components, dicer-like 2 (*dcl2*) and argonaute-like 2 (*agl2*) in *Cryphonectria parasitica* (Andika et al., 2017). We attempted to identify TFs that involved in transcriptional regulation of *FgDICER*s or *FgAGO*s genes using TF deletion mutant library, however, FgV1 was not suitable for screening candidate genes because of the presence of pORF2, suppressor of RNAi (Yu et al., 2020). Further investigations are in progress to identify TFs that play roles in regulating gene expressions of *FgDICER*s or *FgAGO*s combined with present research and other FgV-infected TF deletion mutants.

As mentioned earlier, we had failed to transmit FgV1 *via* hyphal anastomosis into several TF deletion mutants including Δ*GzZC030* (FGSG_06380), Δ*GzZC032* (FGSG_00153), Δ*GzZC044* (FGSG_12094), Δ*GzZC060* (FGSG_08808), Δ*GzZC232* (FGSG_07067), Δ*FgArt1* (FGSG_02083), Δ*GzZC301* (FGSG_00404), and Δ*GzZC316* (FGSG_00125). Most of those gene deletion mutants did not show specific alteration in mycelial growth. Among them, we confirmed gene expression levels of 9 TFs by qRT-PCR (Supplementary Table 4). Seven out of nine genes showed significant changes of gene expression levels following FgV1 infection. Some of these genes might be involved in cell-to-cell interaction regulation that has been proposed as a defense mechanism of host fungi to limit the transmission of mycoviruses (Nuss, 2011). For example, *GzZC232* encoded protein shares 59% sequence identity with *Epichloë festucae* ProA, which is similar to *N. crassa* ADV-1 and *Sordaria macrospora* Pro1 (Tanaka et al., 2013). ProA deletion mutant is defective in hyphal fusion under nutrient limitation condition (Tanaka et al., 2013). In case of *FgArt1*, it is associated with biosynthesis of trichothecene and fumonisin by regulating genes involved in starch hydrolysis, however, it remains unclear if *FgArt1* plays a role in cell fusion or related biological processes (Oh et al., 2016). Many genes and molecular signaling networks are involved during hyphal fusion in *N. crassa* including MAPKinase cascades, a STRIPAK complex, transcription factors, a NADPH-oxidases complex, ROS systems, and Ca^2+^-binding regulators (Fischer and Glass, 2019). Further detailed study is required to explain this inability of hyphal fusion in some TF deletion mutants in *F. graminearum.*

Kinases and phosphatases also contribute to the regulation of gene expression by interacting with transcription factors (Ariño et al., 2019; González-Rubio et al., 2019). Both phosphatidylinositol-3-kinase (PI3K) and Akt signaling pathways promote viral replication and activate antiviral response (Dunn and Connor, 2012). Since systematic characterization of the kinome and phosphatome has been reported in *F. graminearum* previously (Wang et al., 2011; Yun et al., 2015), applying FgV1 into kinome and phosphatome in *F. graminearum* will provide a valuable resource to understand fungal host cell signaling pathway involved in antiviral or proviral functions. Although the TF phenome data illustrated characteristics of phenotype of all TF deletion mutants in previous research, it has limitation in expecting possible functions of TF genes that do not show distinct phenotypic change. In this regard, FgV1-infected TF deletion mutant library obtained in present study would provide chance to better characterize function(s) of novel TF genes that showed distinguishable phenotypes following FgV1-infection. Further study will explore the roles of these TF genes and their putative target genes during FgV1 infection.

## Data Availability Statement

The raw data supporting the conclusions of this article will be made available by the authors, without undue reservation.

## Author Contributions

JY and K-HK designed the experiments, analyzed the data, and wrote the manuscript. JY performed the experimental work. Both authors contributed to the article and approved the submitted version.

## Conflict of Interest

The authors declare that the research was conducted in the absence of any commercial or financial relationships that could be construed as a potential conflict of interest.
