# Supplementary material for: A Phenome-Wide Association Study of the Effects of Fusarium graminearum Transcription Factors on Fusarium Graminearum Virus 1 Infection
Source: Front Microbiol. 2021 Feb 11;12:622261. doi: 10.3389/fmicb.2021.622261 (PMC7904688; doi:10.3389/fmicb.2021.622261)
Supplement: Supplementary file 1 [file Data_Sheet_1.pdf]

Supplementary Figure 1. Generation of FgV1-infected TF gene-deletion mutants of *Fusarium graminearum*. Colony morphology of FgV1-infected TF deletion mutants are shown**.** All virus-free and FgV1-infected cultures were photographed after 5 days on complete medium. Most of virus-free TF deletion mutants that did not changed mycelial growth was not photographed in present study.

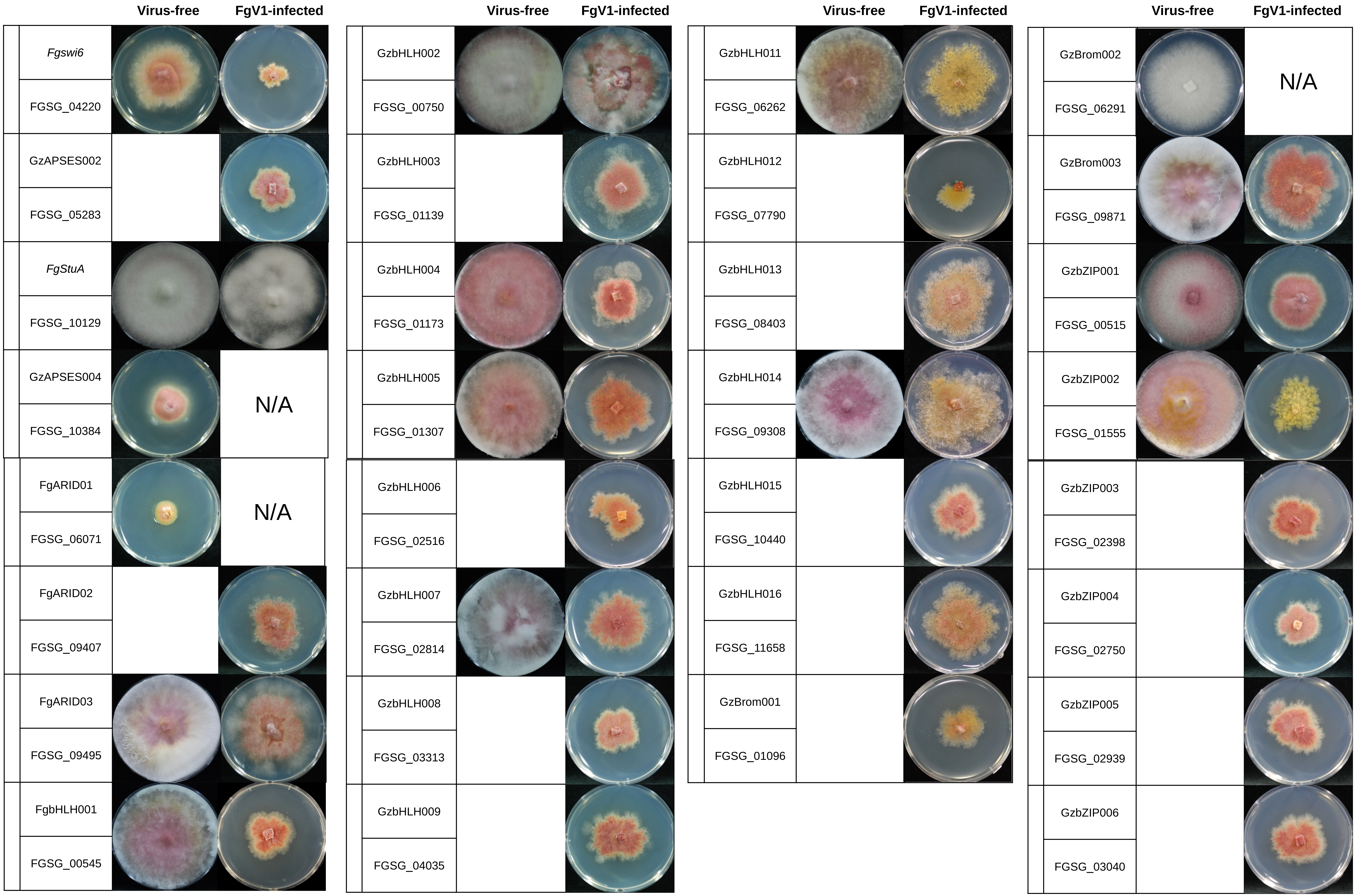

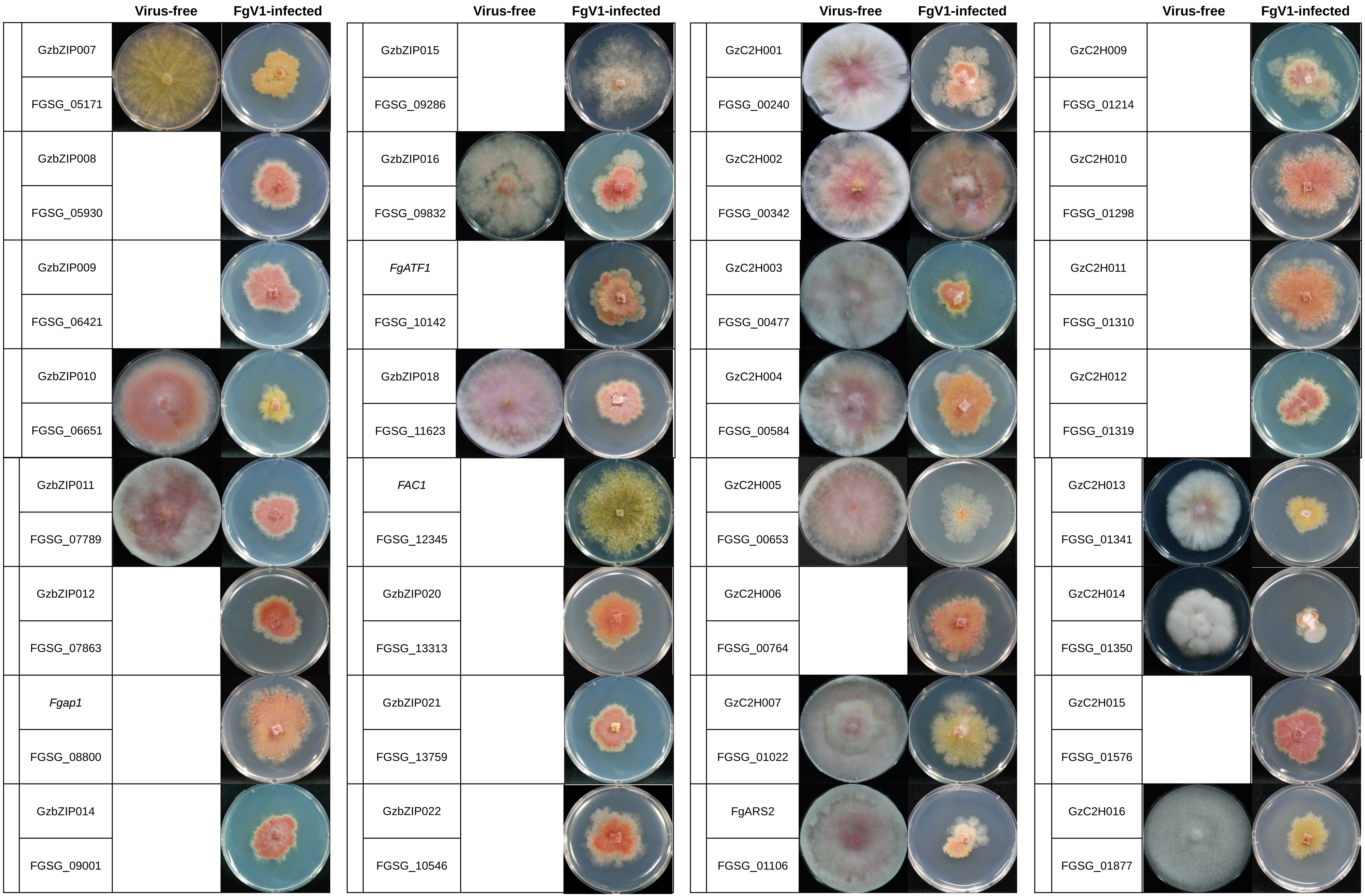
Virus-free
FgV1-infected
GzbZIP007
FGSG_05171
GzbZIP008
FGSG_05930
GzbZIP009
FGSG_06421
GzbZIP010
FGSG_06651
GzbZIP011
FGSG_07789
GzbZIP012
FGSG_07863
Fgap1
FGSG_08800
GzbZIP014
FGSG_09001
GzbZIP015
FGSG_09286
GzbZIP016
FGSG_09832
FgATF1
FGSG_10142
GzbZIP018
FGSG_11623
FAC1
FGSG_12345
GzbZIP020
FGSG_13313
GzbZIP021
FGSG_13759
GzbZIP022
FGSG_10546
GzC2H001
FGSG_00240
GzC2H002
FGSG_00342
GzC2H003
FGSG_00477
GzC2H004
FGSG_00584
GzC2H005
FGSG_00653
GzC2H006
FGSG_00764
GzC2H007
FGSG_01022
FgARS2
FGSG_01106
GzC2H009
FGSG_01214
GzC2H010
FGSG_01298
GzC2H011
FGSG_01310
GzC2H012
FGSG_01319
GzC2H013
FGSG_01341
GzC2H014
FGSG_01350
GzC2H015
FGSG_01576
GzC2H016
FGSG_01877

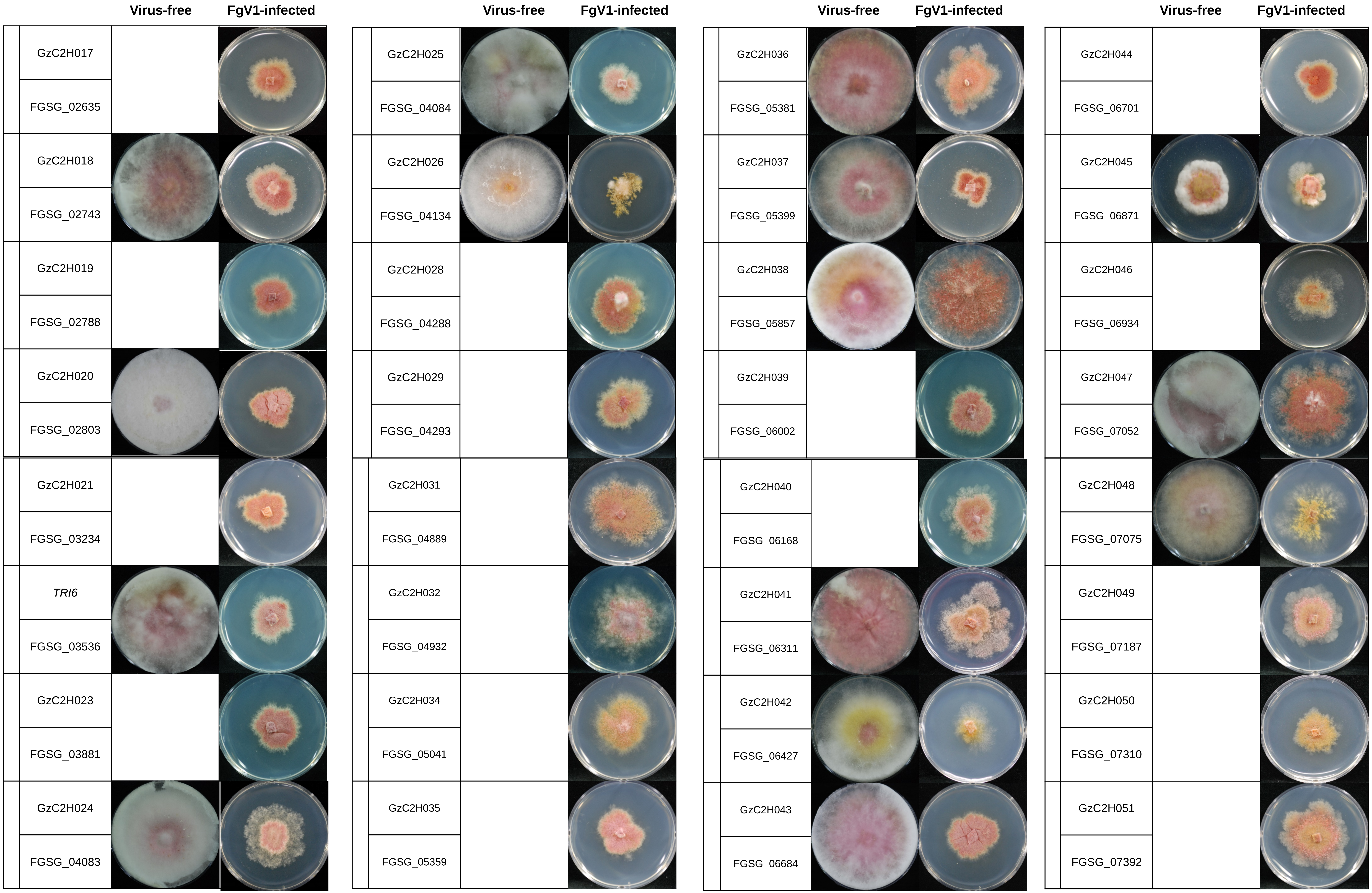
Virus-free
FgV1-infected
GzC2H017
FGSG_02635
GzC2H018
FGSG_02743
GzC2H019
FGSG_02788
GzC2H020
FGSG_02803
GzC2H021
FGSG_03234
TRI6
FGSG_03536
GzC2H023
FGSG_03881
GzC2H024
FGSG_04083
Virus-free
FgV1-infected
GzC2H025
FGSG_04084
GzC2H026
FGSG_04134
GzC2H028
FGSG_04288
GzC2H029
FGSG_04293
GzC2H031
FGSG_04889
GzC2H032
FGSG_04932
GzC2H034
FGSG_05041
GzC2H035
FGSG_05359
Virus-free
FgV1-infected
GzC2H036
FGSG_05381
GzC2H037
FGSG_05399
GzC2H038
FGSG_05857
GzC2H039
FGSG_06002
GzC2H040
FGSG_06168
GzC2H041
FGSG_06311
GzC2H042
FGSG_06427
GzC2H043
FGSG_06684
Virus-free
FgV1-infected
GzC2H044
FGSG_06701
GzC2H045
FGSG_06871
GzC2H046
FGSG_06934
GzC2H047
FGSG_07052
GzC2H048
FGSG_07075
GzC2H049
FGSG_07187
GzC2H050
FGSG_07310
GzC2H051
FGSG_07392

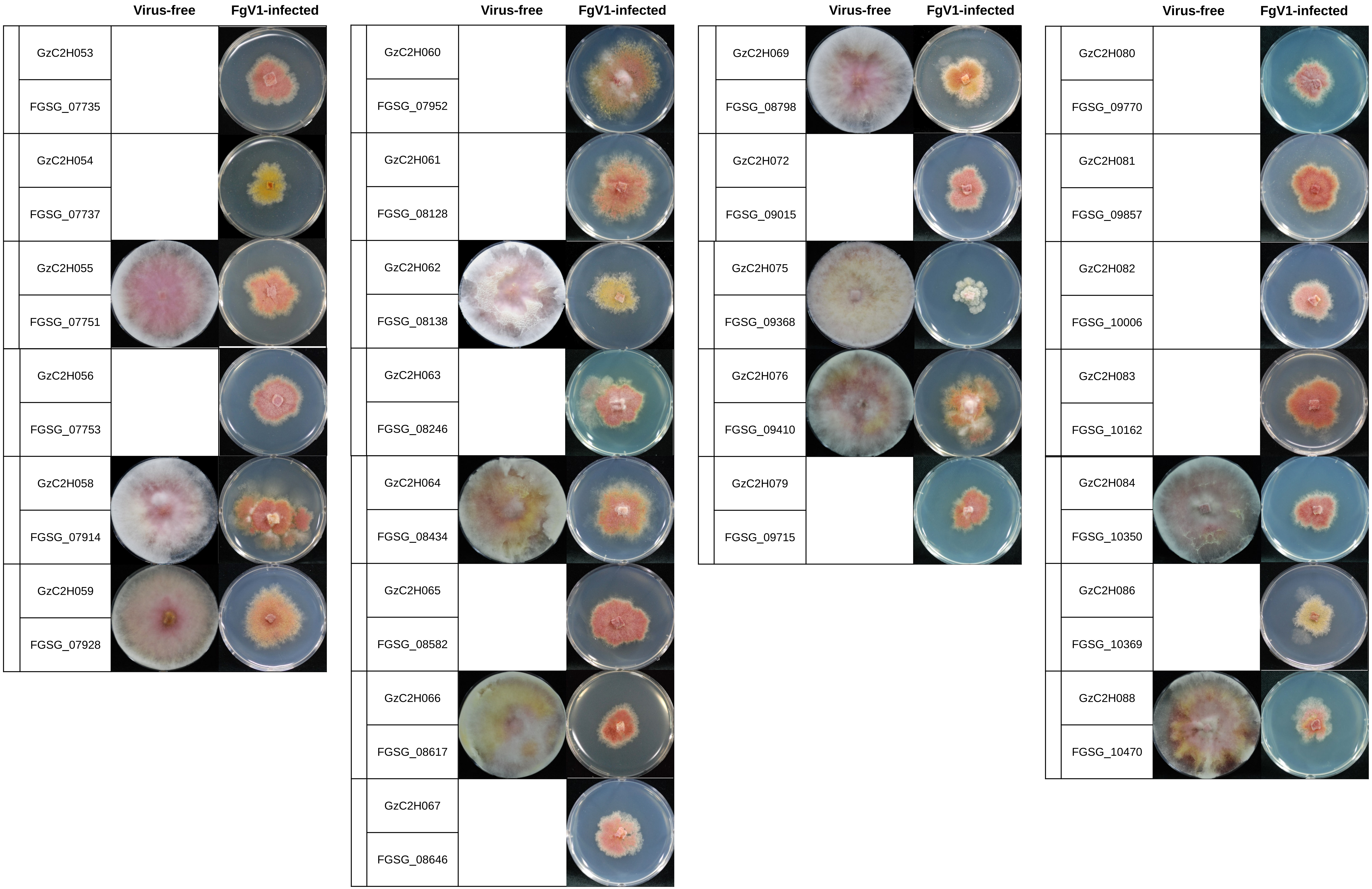
Virus-free
FgV1-infected
GzC2H053
FGSG_07735
GzC2H054
FGSG_07737
GzC2H055
FGSG_07751
GzC2H056
FGSG_07753
GzC2H058
FGSG_07914
GzC2H059
FGSG_07928
Virus-free
FgV1-infected
GzC2H060
FGSG_07952
GzC2H061
FGSG_08128
GzC2H062
FGSG_08138
GzC2H063
FGSG_08246
GzC2H064
FGSG_08434
GzC2H065
FGSG_08582
GzC2H066
FGSG_08617
GzC2H067
FGSG_08646
Virus-free
FgV1-infected
GzC2H069
FGSG_08798
GzC2H072
FGSG_09015
GzC2H075
FGSG_09368
GzC2H076
FGSG_09410
GzC2H079
FGSG_09715
Virus-free
FgV1-infected
GzC2H080
FGSG_09770
GzC2H081
FGSG_09857
GzC2H082
FGSG_10006
GzC2H083
FGSG_10162
GzC2H084
FGSG_10350
GzC2H086
FGSG_10369
GzC2H088
FGSG_10470

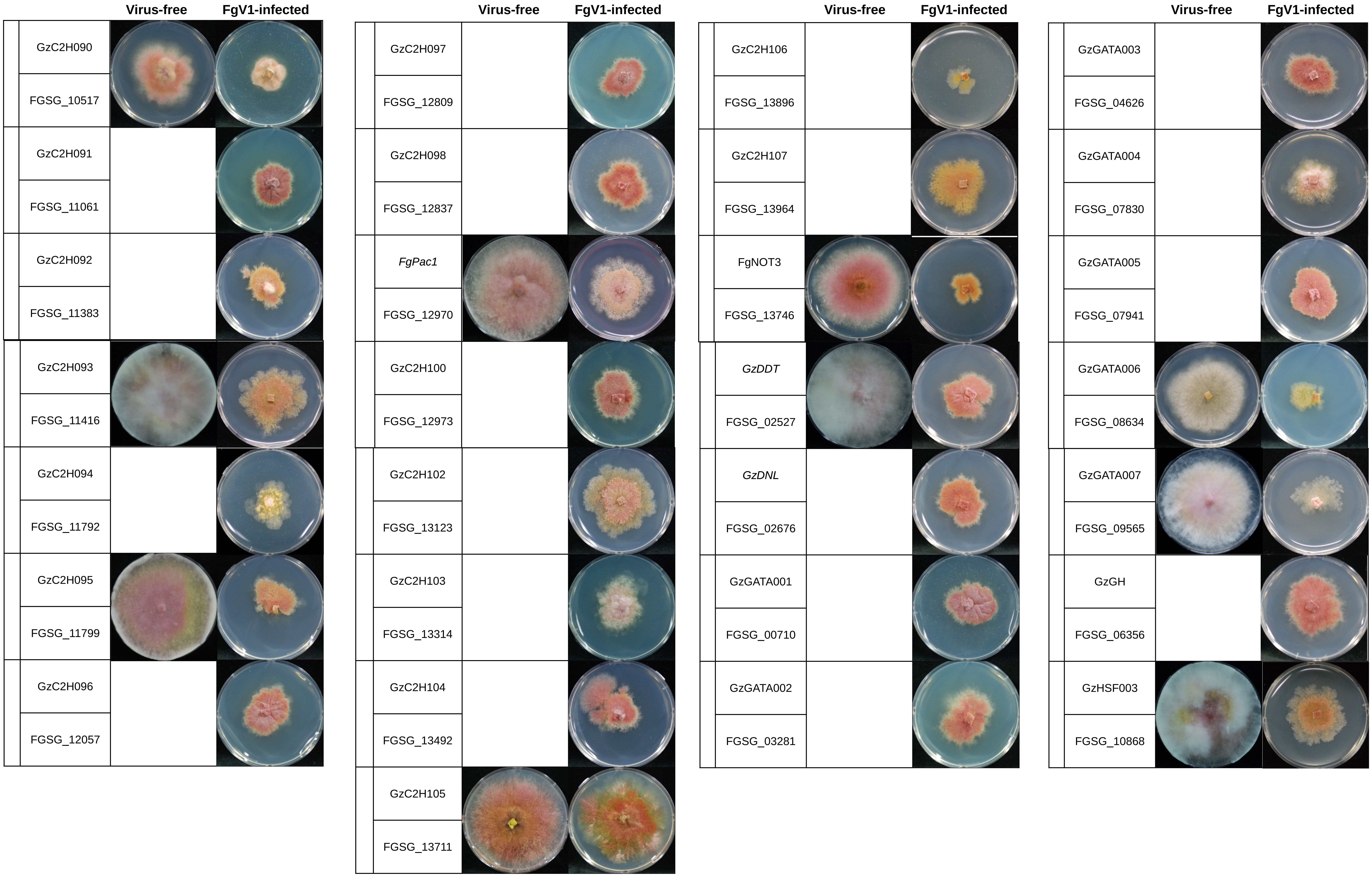
Virus-free
FgV1-infected
GzC2H090
FGSG_10517
GzC2H091
FGSG_11061
GzC2H092
FGSG_11383
GzC2H093
FGSG_11416
GzC2H094
FGSG_11792
GzC2H095
FGSG_11799
GzC2H096
FGSG_12057
Virus-free
FgV1-infected
GzC2H097
FGSG_12809
GzC2H098
FGSG_12837
FgPac1
FGSG_12970
GzC2H100
FGSG_12973
GzC2H102
FGSG_13123
GzC2H103
FGSG_13314
GzC2H104
FGSG_13492
GzC2H105
FGSG_13711
Virus-free
FgV1-infected
GzC2H106
FGSG_13896
GzC2H107
FGSG_13964
FgNOT3
FGSG_13746
GzDDT
FGSG_02527
GzDNL
FGSG_02676
GzGATA001
FGSG_00710
GzGATA002
FGSG_03281
Virus-free
FgV1-infected
GzGATA003
FGSG_04626
GzGATA004
FGSG_07830
GzGATA005
FGSG_07941
GzGATA006
FGSG_08634
GzGATA007
FGSG_09565
GzGH
FGSG_06356
GzHSF003
FGSG_10868

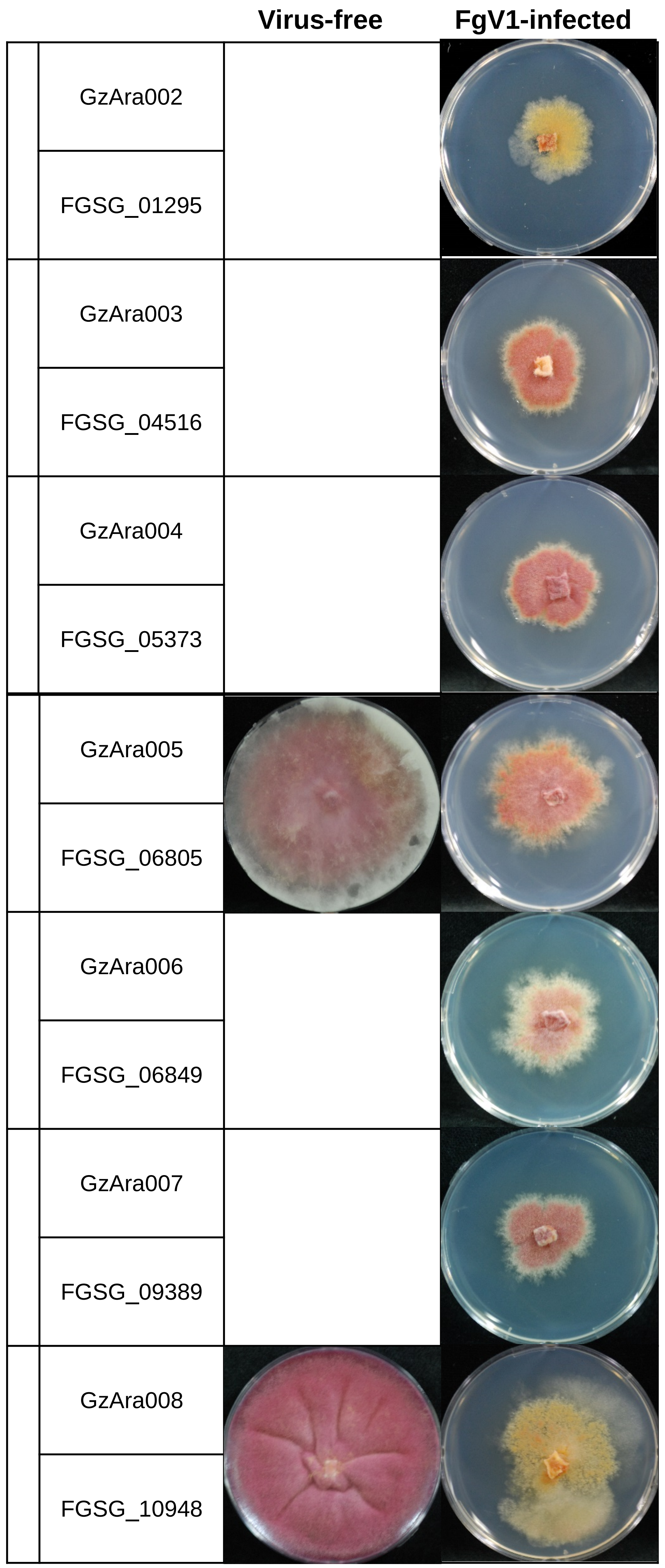
Virus-free
FgV1-infected
GzAra002
FGSG_01295
GzAra003
FGSG_04516
GzAra004
FGSG_05373
GzAra005
FGSG_06805
GzAra006
FGSG_06849
GzAra007
FGSG_09389
GzAra008
FGSG_10948

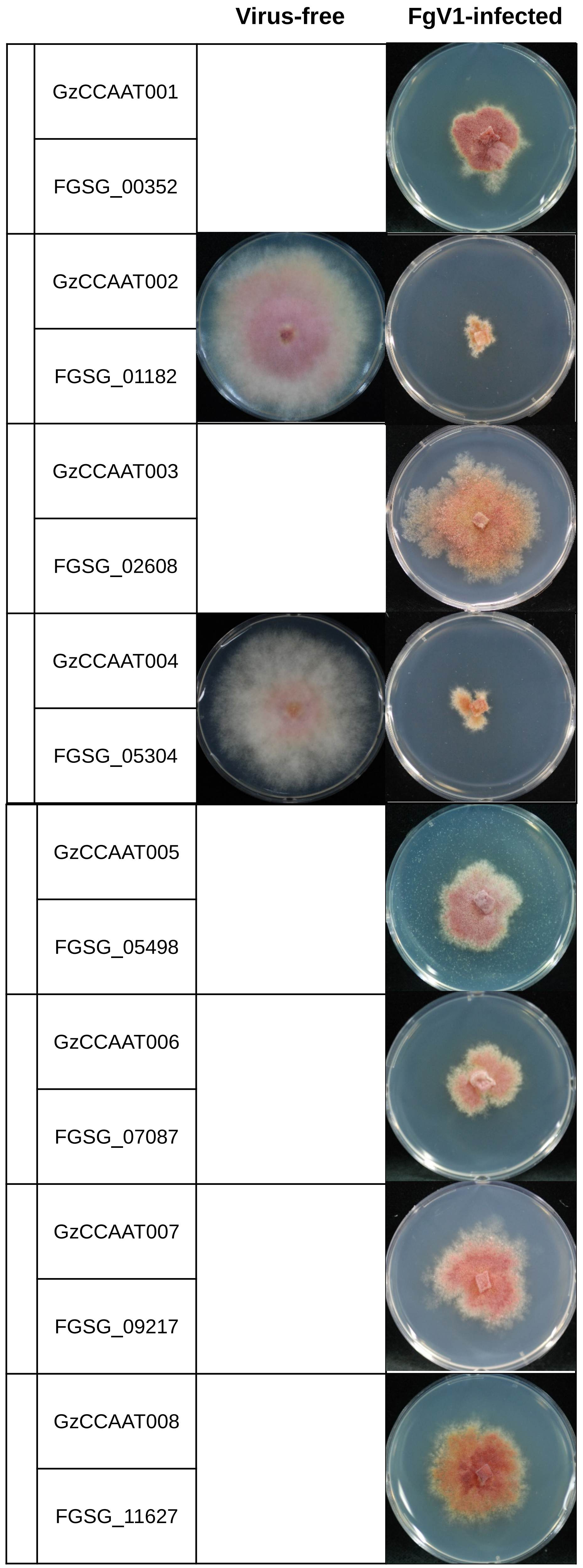
Virus-free
FgV1-infected
GzCCAAT001
FGSG_00352
GzCCAAT002
FGSG_01182
GzCCAAT003
FGSG_02608
GzCCAAT004
FGSG_05304
GzCCAAT005
FGSG_05498
GzCCAAT006
FGSG_07087
GzCCAAT007
FGSG_09217
GzCCAAT008
FGSG_11627

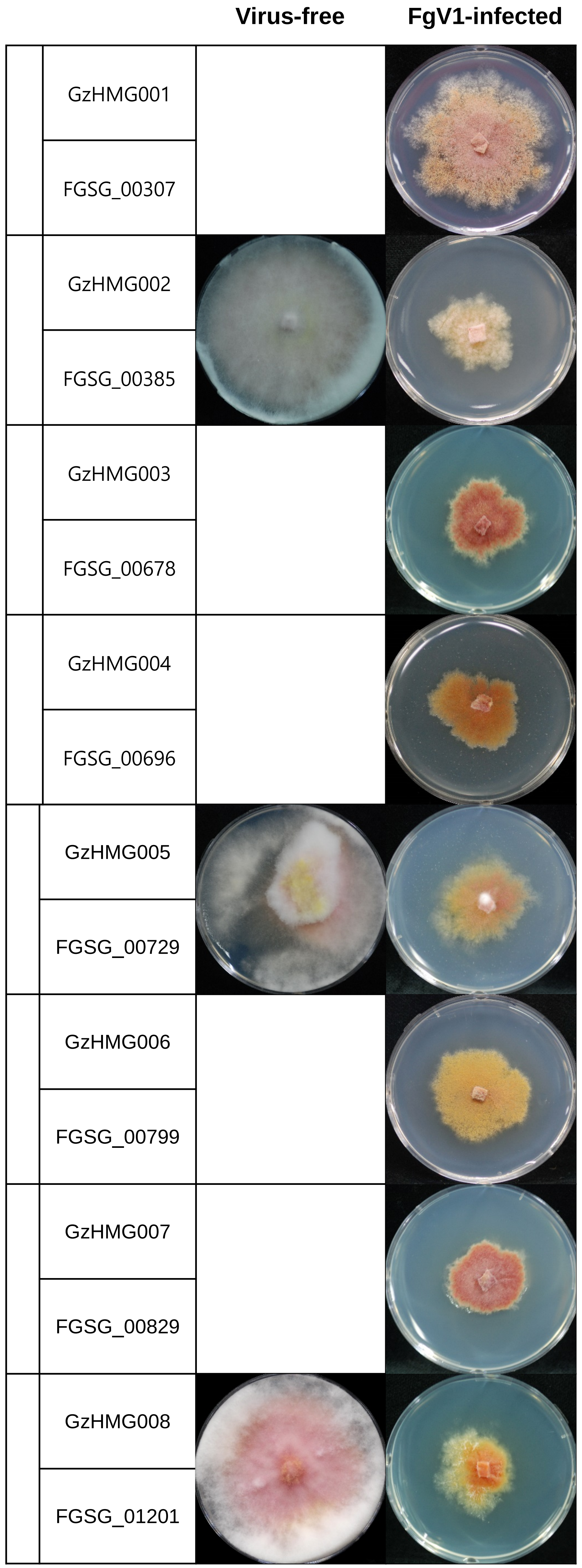
Virus-free
FgV1-infected
GzHMG001
FGSG_00307
GzHMG002
FGSG_00385
GzHMG003
FGSG_00678
GzHMG004
FGSG_00696
GzHMG005
FGSG_00729
GzHMG006
FGSG_00799
GzHMG007
FGSG_00829
GzHMG008
FGSG_01201

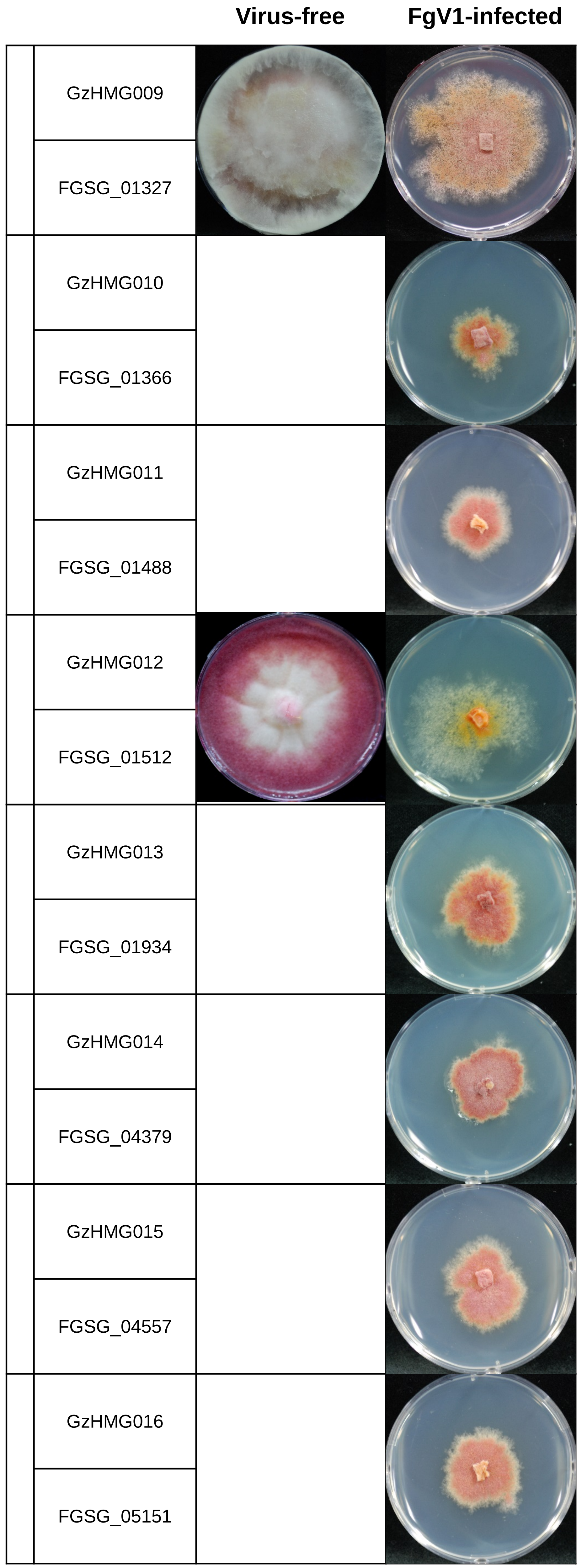
Virus-free
FgV1-infected
GzHMG009
FGSG_01327
GzHMG010
FGSG_01366
GzHMG011
FGSG_01488
GzHMG012
FGSG_01512
GzHMG013
FGSG_01934
GzHMG014
FGSG_04379
GzHMG015
FGSG_04557
GzHMG016
FGSG_05151

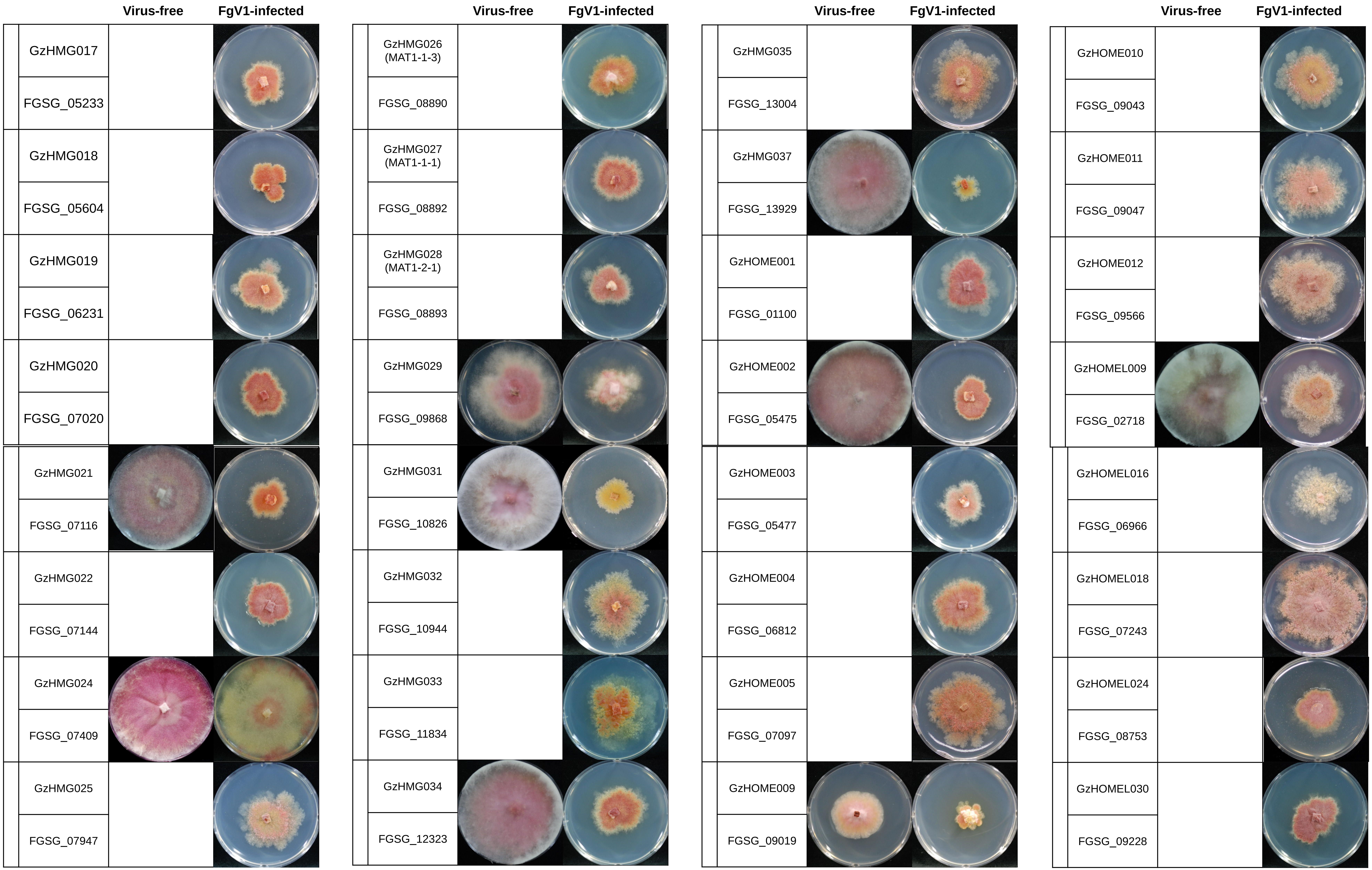

Virus-free
FgV1-infected
GzHMG017
FGSG_05233
GzHMG018
FGSG_05604
GzHMG019
FGSG_06231
GzHMG020
FGSG_07020
GzHMG021
FGSG_07116
GzHMG022
FGSG_07144
GzHMG024
FGSG_07409
GzHMG025
FGSG_07947
Virus-free
FgV1-infected
GzHMG026 (MAT1-1-3)
FGSG_08890
GzHMG027 (MAT1-1-1)
FGSG_08892
GzHMG028 (MAT1-2-1)
FGSG_08893
GzHMG029
FGSG_09868
GzHMG031
FGSG_10826
GzHMG032
FGSG_10944
GzHMG033
FGSG_11834
GzHMG034
FGSG_12323
Virus-free
FgV1-infected
GzHMG035
FGSG_13004
GzHMG037
FGSG_13929
GzHOME001
FGSG_01100
GzHOME002
FGSG_05475
GzHOME003
FGSG_05477
GzHOME004
FGSG_06812
GzHOME005
FGSG_07097
GzHOME009
FGSG_09019
Virus-free
FgV1-infected
GzHOME010
FGSG_09043
GzHOME011
FGSG_09047
GzHOME012
FGSG_09566
GzHOMEL009
FGSG_02718
GzHOMEL016
FGSG_06966
GzHOMEL018
FGSG_07243
GzHOMEL024
FGSG_08753
GzHOMEL030
FGSG_09228

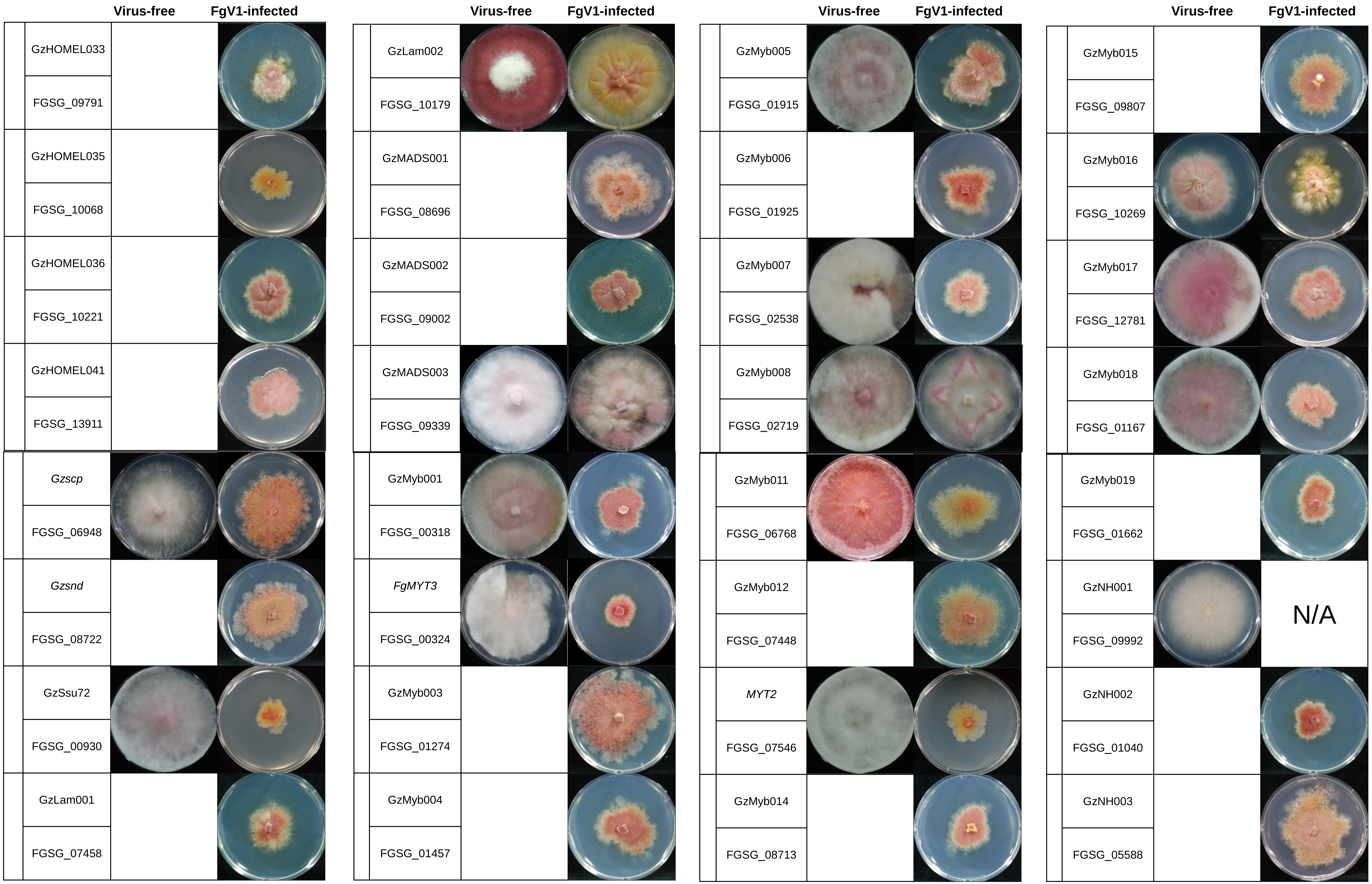
Virus-free
FgV1-infected
GzHOMEL033
FGSG_09791
GzHOMEL035
FGSG_10068
GzHOMEL036
FGSG_10221
GzHOMEL041
FGSG_13911
Gzscp
FGSG_06948
Gzsnd
FGSG_08722
GzSsu72
FGSG_00930
GzLam001
FGSG_07458
Virus-free
FgV1-infected
GzLam002
FGSG_10179
GzMADS001
FGSG_08696
GzMADS002
FGSG_09002
GzMADS003
FGSG_09339
GzMyb001
FGSG_00318
FgMYT3
FGSG_00324
GzMyb003
FGSG_01274
GzMyb004
FGSG_01457
Virus-free
FgV1-infected
GzMyb005
FGSG_01915
GzMyb006
FGSG_01925
GzMyb007
FGSG_02538
GzMyb008
FGSG_02719
GzMyb011
FGSG_06768
GzMyb012
FGSG_07448
MYT2
FGSG_07546
GzMyb014
FGSG_08713
Virus-free
FgV1-infected
GzMyb015
FGSG_09807
GzMyb016
FGSG_10269
GzMyb017
FGSG_12781
GzMyb018
FGSG_01167
GzMyb019
FGSG_01662
GzNH001
FGSG_09992
N/A
GzNH002
FGSG_01040
GzNH003
FGSG_05588

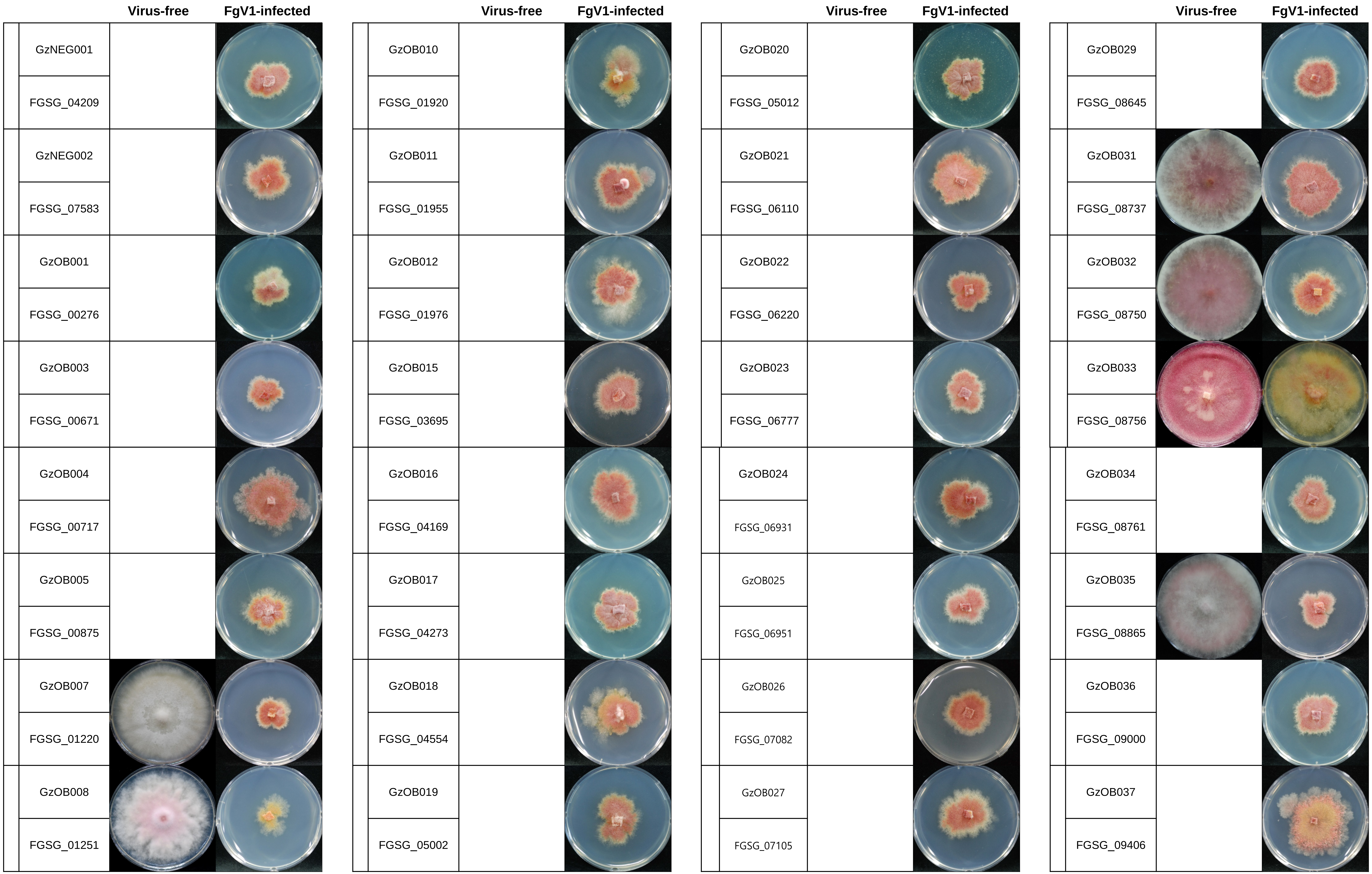
Virus-free
FgV1-infected
GzNEG001
FGSG_04209
GzNEG002
FGSG_07583
GzOB001
FGSG_00276
GzOB003
FGSG_00671
GzOB004
FGSG_00717
GzOB005
FGSG_00875
GzOB007
FGSG_01220
GzOB008
FGSG_01251
Virus-free
FgV1-infected
GzOB010
FGSG_01920
GzOB011
FGSG_01955
GzOB012
FGSG_01976
GzOB015
FGSG_03695
GzOB016
FGSG_04169
GzOB017
FGSG_04273
GzOB018
FGSG_04554
GzOB019
FGSG_05002
Virus-free
FgV1-infected
GzOB020
FGSG_05012
GzOB021
FGSG_06110
GzOB022
FGSG_06220
GzOB023
FGSG_06777
GzOB024
FGSG_06931
GzOB025
FGSG_06951
GzOB026
FGSG_07082
GzOB027
FGSG_07105
Virus-free
FgV1-infected
GzOB029
FGSG_08645
GzOB031
FGSG_08737
GzOB032
FGSG_08750
GzOB033
FGSG_08756
GzOB034
FGSG_08761
GzOB035
FGSG_08865
GzOB036
FGSG_09000
GzOB037
FGSG_09406

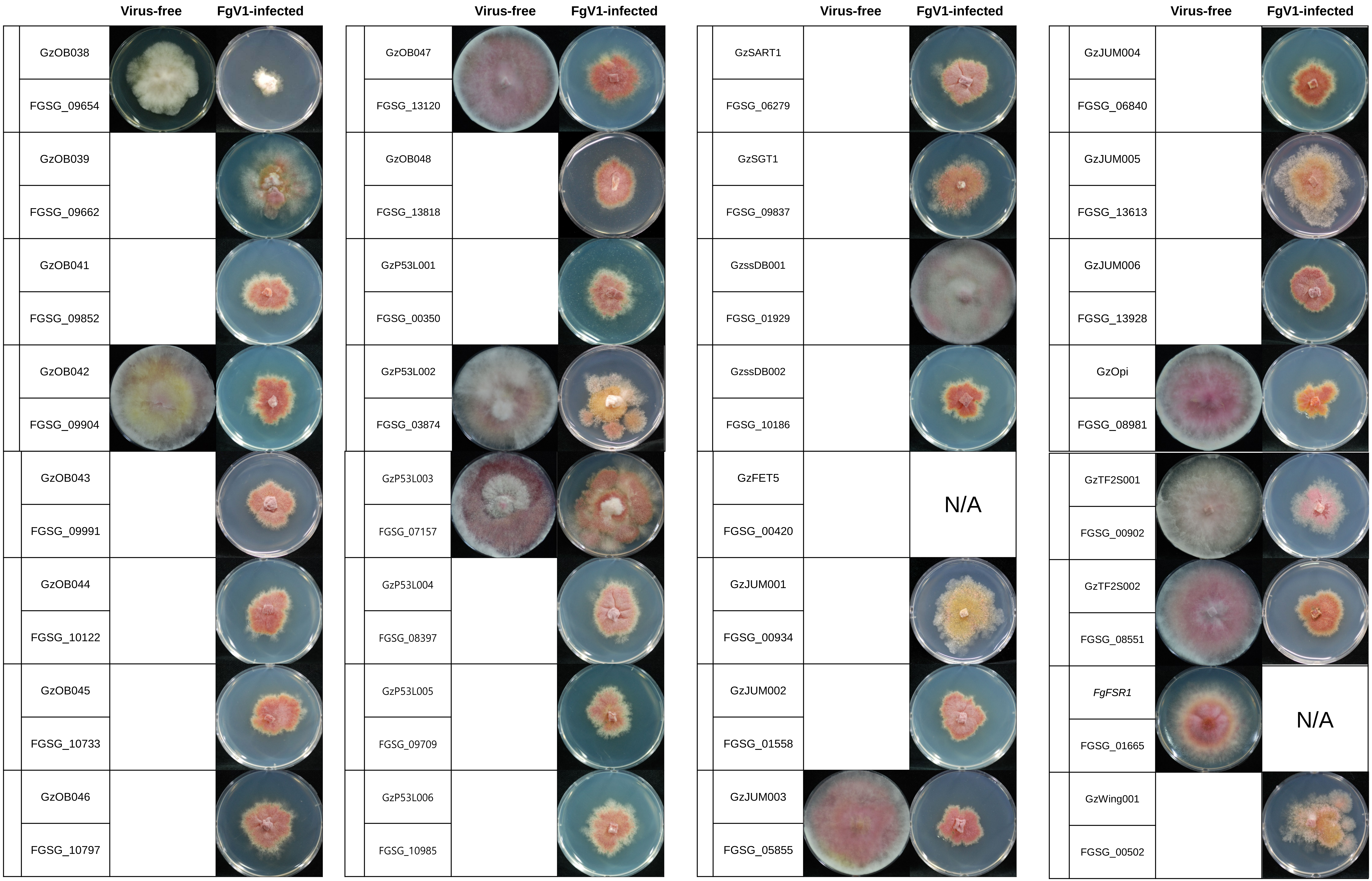
Virus-free
FgV1-infected
GzOB038
FGSG_09654
GzOB039
FGSG_09662
GzOB041
FGSG_09852
GzOB042
FGSG_09904
GzOB043
FGSG_09991
GzOB044
FGSG_10122
GzOB045
FGSG_10733
GzOB046
FGSG_10797
Virus-free
FgV1-infected
GzOB047
FGSG_13120
GzOB048
FGSG_13818
GzP53L001
FGSG_00350
GzP53L002
FGSG_03874
GzP53L003
FGSG_07157
GzP53L004
FGSG_08397
GzP53L005
FGSG_09709
GzP53L006
FGSG_10985
Virus-free
FgV1-infected
GzSART1
FGSG_06279
GzSGT1
FGSG_09837
GzssDB001
FGSG_01929
GzssDB002
FGSG_10186
GzFET5
FGSG_00420
N/A
GzJUM001
FGSG_00934
GzJUM002
FGSG_01558
GzJUM003
FGSG_05855
Virus-free
FgV1-infected
GzJUM004
FGSG_06840
GzJUM005
FGSG_13613
GzJUM006
FGSG_13928
GzOpi
FGSG_08981
GzTF2S001
FGSG_00902
GzTF2S002
FGSG_08551
FgFSR1
FGSG_01665
N/A
GzWing001
FGSG_00502

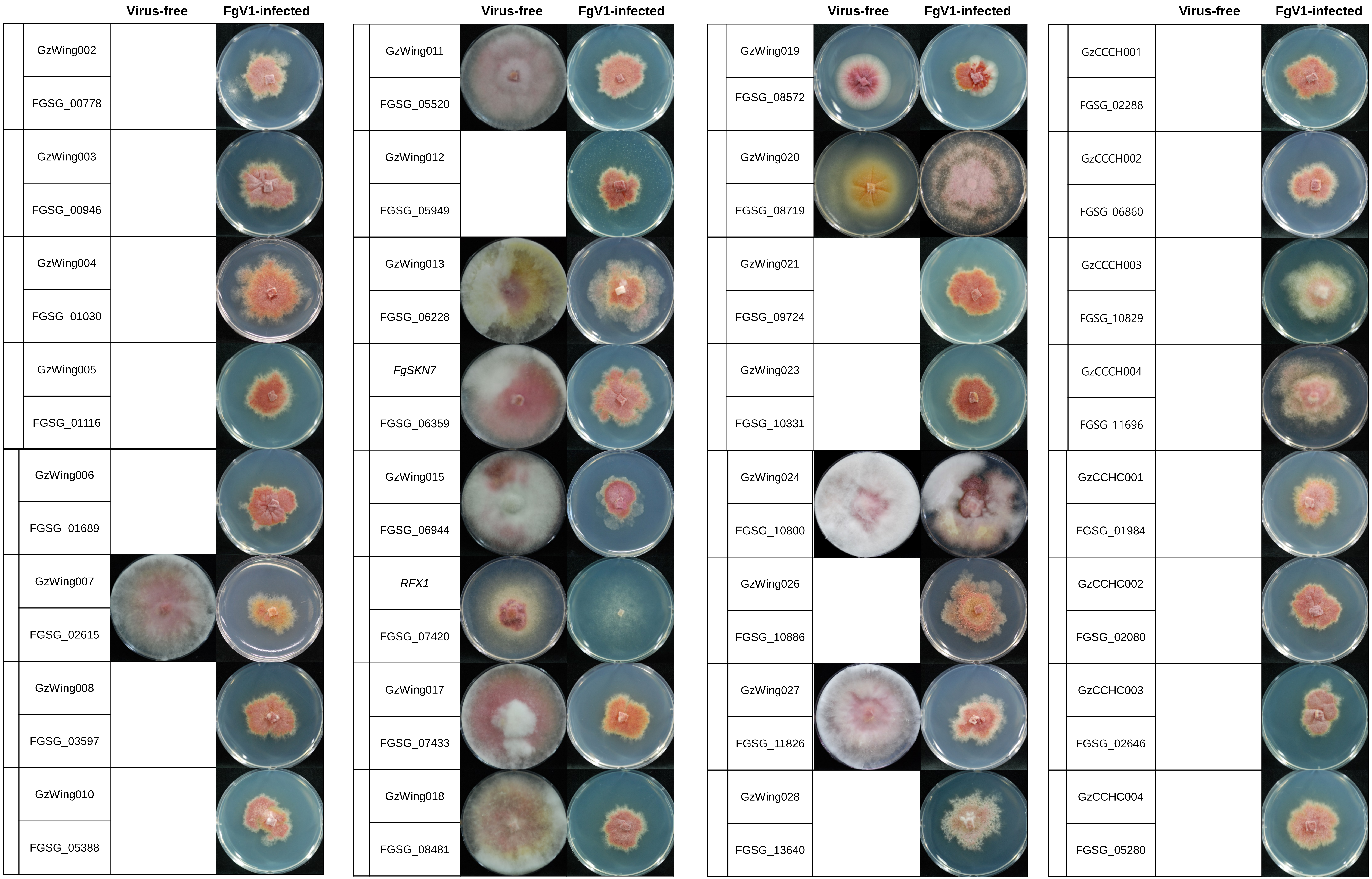
Virus-free
FgV1-infected
GzWing002
FGSG_00778
GzWing003
FGSG_00946
GzWing004
FGSG_01030
GzWing005
FGSG_01116
GzWing006
FGSG_01689
GzWing007
FGSG_02615
GzWing008
FGSG_03597
GzWing010
FGSG_05388
Virus-free
FgV1-infected
GzWing011
FGSG_05520
GzWing012
FGSG_05949
GzWing013
FGSG_06228
FgSKN7
FGSG_06359
GzWing015
FGSG_06944
RFX1
FGSG_07420
GzWing017
FGSG_07433
GzWing018
FGSG_08481
Virus-free
FgV1-infected
GzWing019
FGSG_08572
GzWing020
FGSG_08719
GzWing021
FGSG_09724
GzWing023
FGSG_10331
GzWing024
FGSG_10800
GzWing026
FGSG_10886
GzWing027
FGSG_11826
GzWing028
FGSG_13640
Virus-free
FgV1-infected
GzCCCH001
FGSG_02288
GzCCCH002
FGSG_06860
GzCCCH003
FGSG_10829
GzCCCH004
FGSG_11696
GzCCHC001
FGSG_01984
GzCCHC002
FGSG_02080
GzCCHC003
FGSG_02646
GzCCHC004
FGSG_05280

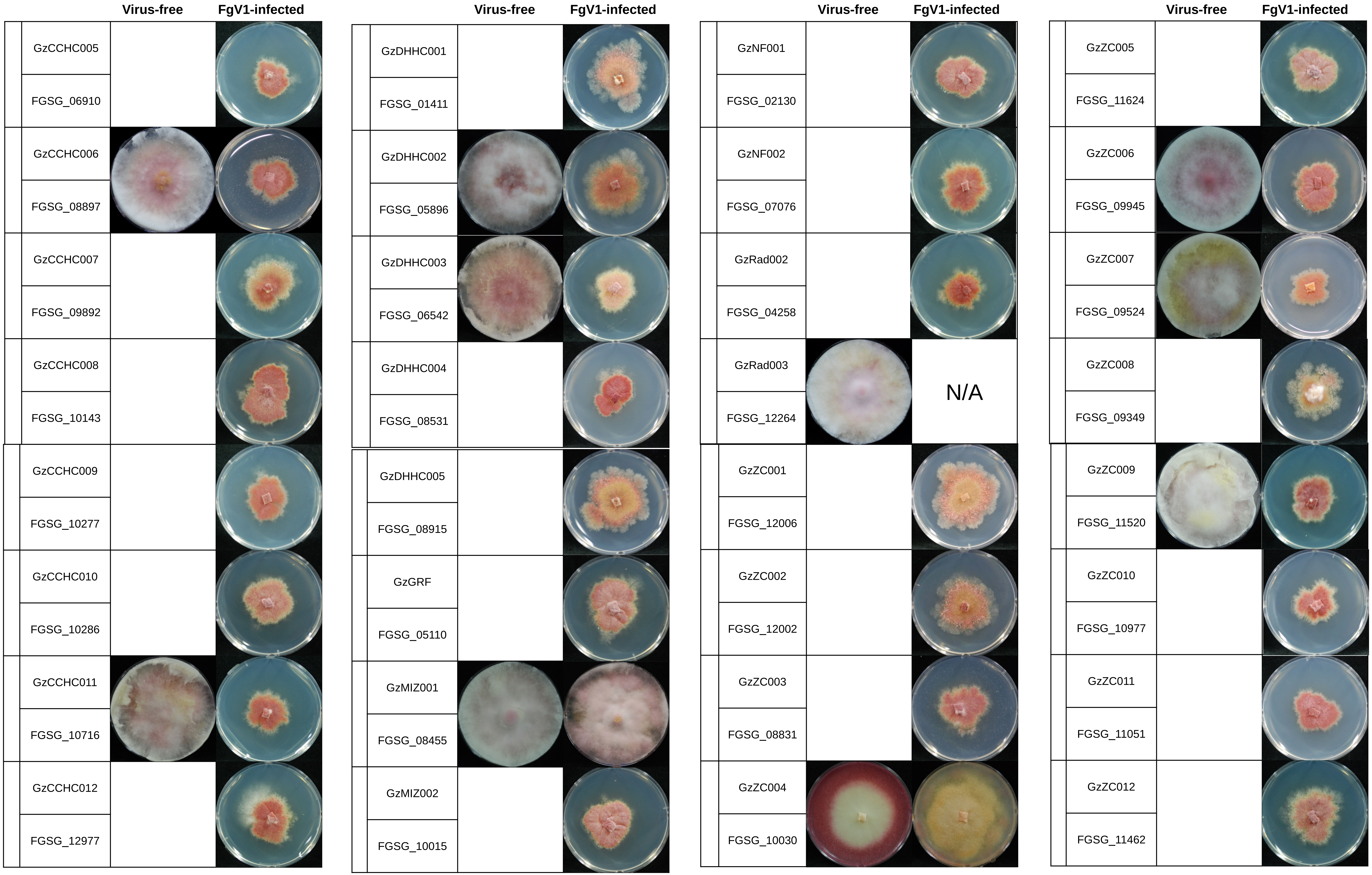
Virus-free
FgV1-infected
GzCCHC005
FGSG_06910
GzCCHC006
FGSG_08897
GzCCHC007
FGSG_09892
GzCCHC008
FGSG_10143
GzCCHC009
FGSG_10277
GzCCHC010
FGSG_10286
GzCCHC011
FGSG_10716
GzCCHC012
FGSG_12977
Virus-free
FgV1-infected
GzDHHC001
FGSG_01411
GzDHHC002
FGSG_05896
GzDHHC003
FGSG_06542
GzDHHC004
FGSG_08531
GzDHHC005
FGSG_08915
GzGRF
FGSG_05110
GzMIZ001
FGSG_08455
GzMIZ002
FGSG_10015
Virus-free
FgV1-infected
GzNF001
FGSG_02130
GzNF002
FGSG_07076
GzRad002
FGSG_04258
GzRad003
FGSG_12264
N/A
GzZC001
FGSG_12006
GzZC002
FGSG_12002
GzZC003
FGSG_08831
GzZC004
FGSG_10030
Virus-free
FgV1-infected
GzZC005
FGSG_11624
GzZC006
FGSG_09945
GzZC007
FGSG_09524
GzZC008
FGSG_09349
GzZC009
FGSG_11520
GzZC010
FGSG_10977
GzZC011
FGSG_11051
GzZC012
FGSG_11462

	Virus-free	FgV1-infected
GzZC013		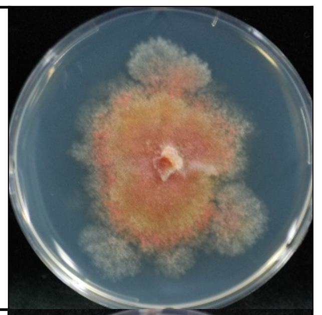
FGSG_10891		
GzZC014		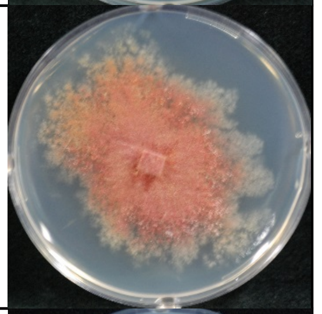
FGSG_04680		
GzZC015		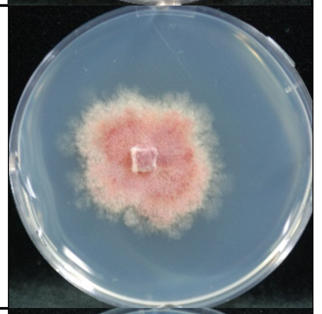
FGSG_04666		
GzZC016		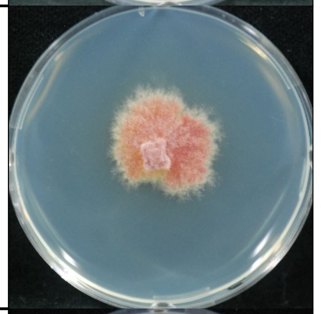
FGSG_04496		
GzZC017		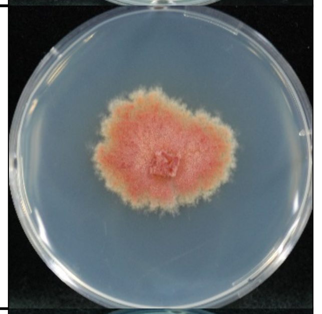
FGSG_07575		
GzZC018		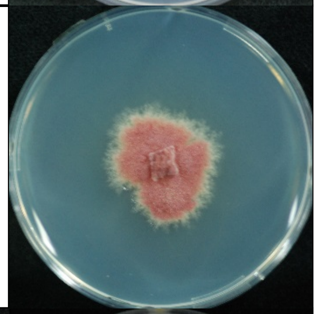
FGSG_07568		
GzZC019	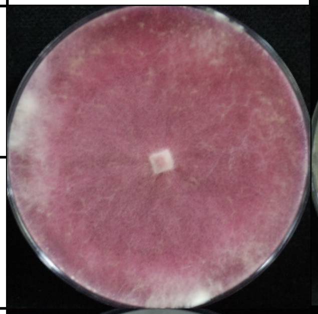	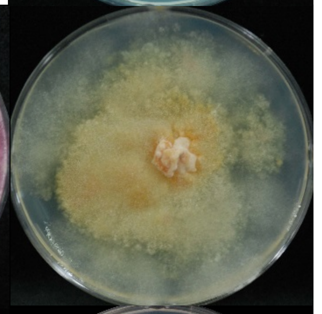
FGSG_05926		
GzZC020	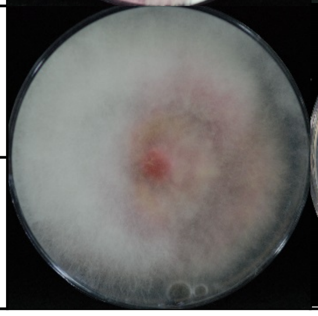	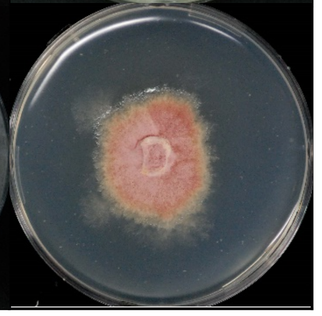
FGSG_03924		

	Virus-free	FgV1-infected
GzZC021		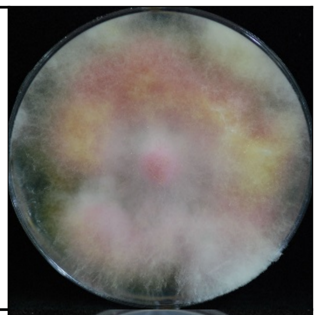
FGSG_03873		
GzZC022		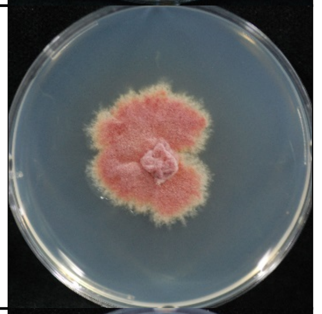
FGSG_04170		
GzZC023		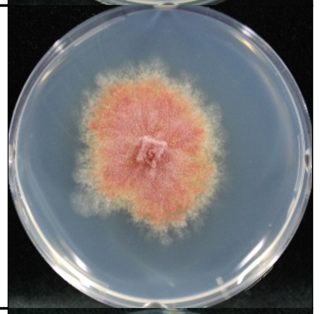
FGSG_05827		
GzZC024		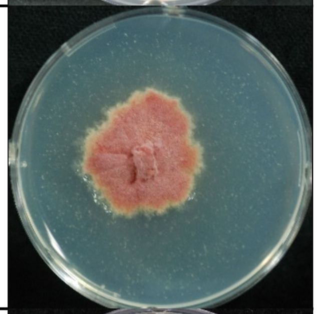
FGSG_06516		
GzZC025		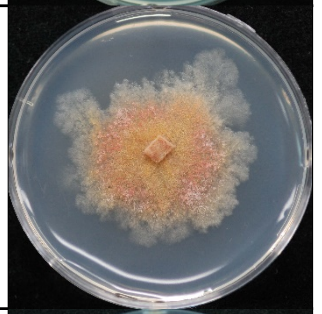
FGSG_03606		
GzZC027		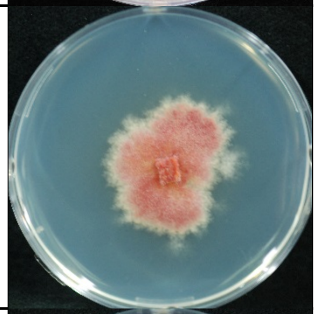
FGSG_05309		
GzZC028		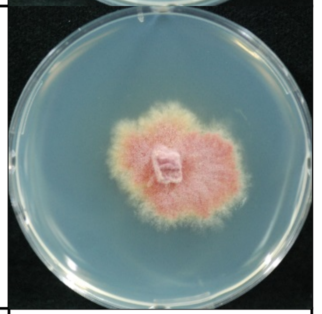
FGSG_02825		
GzZC030		N/A
FGSG_06380		

	Virus-free	FgV1-infected
GzZC031		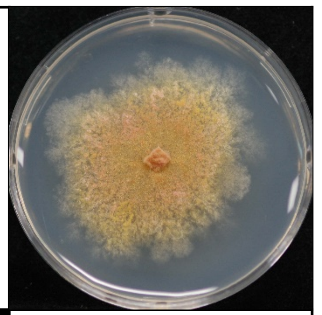
FGSG_04782		
GzZC032	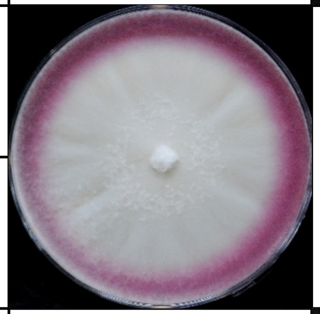	N/A
FGSG_00153		
GzZC033		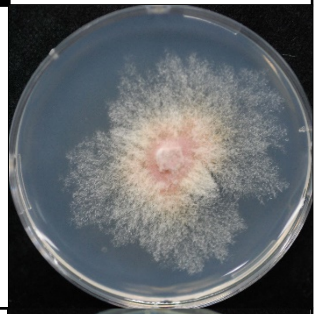
FGSG_13652		
GzZC034		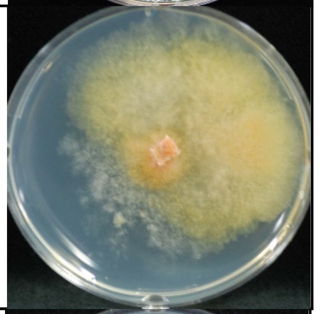
FGSG_13625		
GzZC035		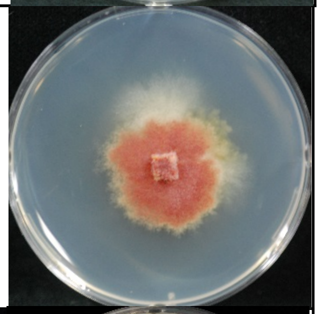
FGSG_13386		
GzZC036	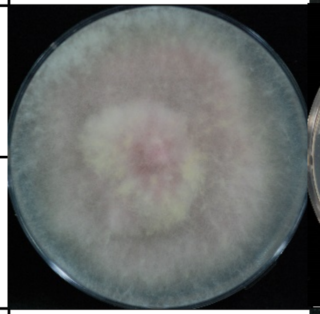	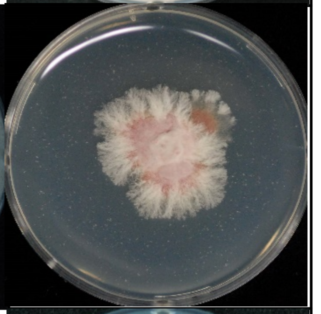
FGSG_13344		
GzZC037		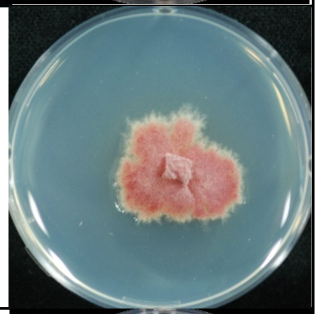
FGSG_13098		
GzZC038		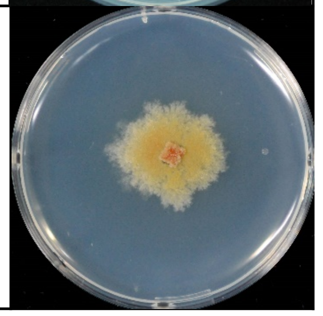
FGSG_13008		

	Virus-free	FgV1-infected
GzZC039		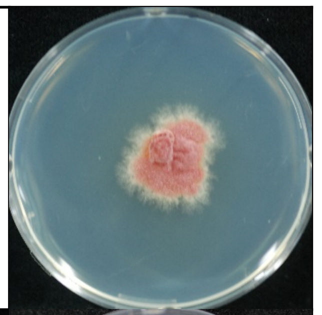
FGSG_13830		
GzZC040	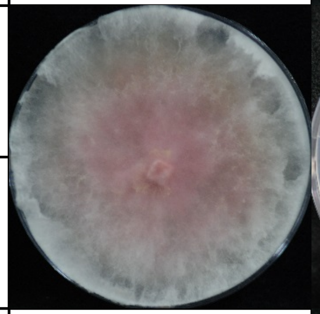	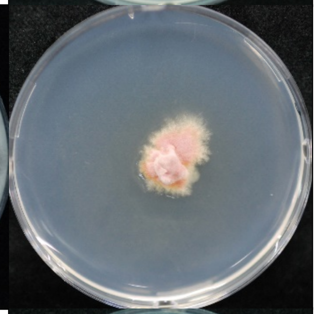
FGSG_13828		
GzZC041		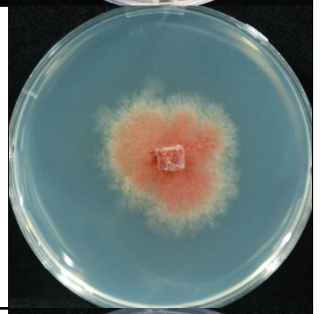
FGSG_13457		
GzZC042		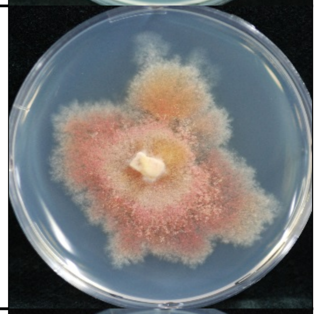
FGSG_12173		
GzZC043		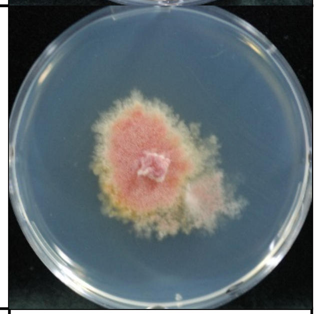
FGSG_12134		
GzZC044		N/A
FGSG_12094		
GzZC045		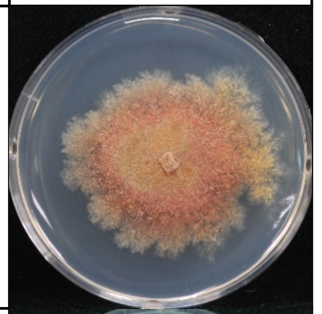
FGSG_12398		
GzZC046		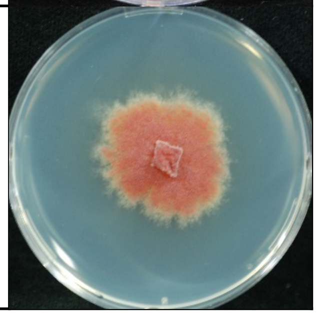
FGSG_11996		

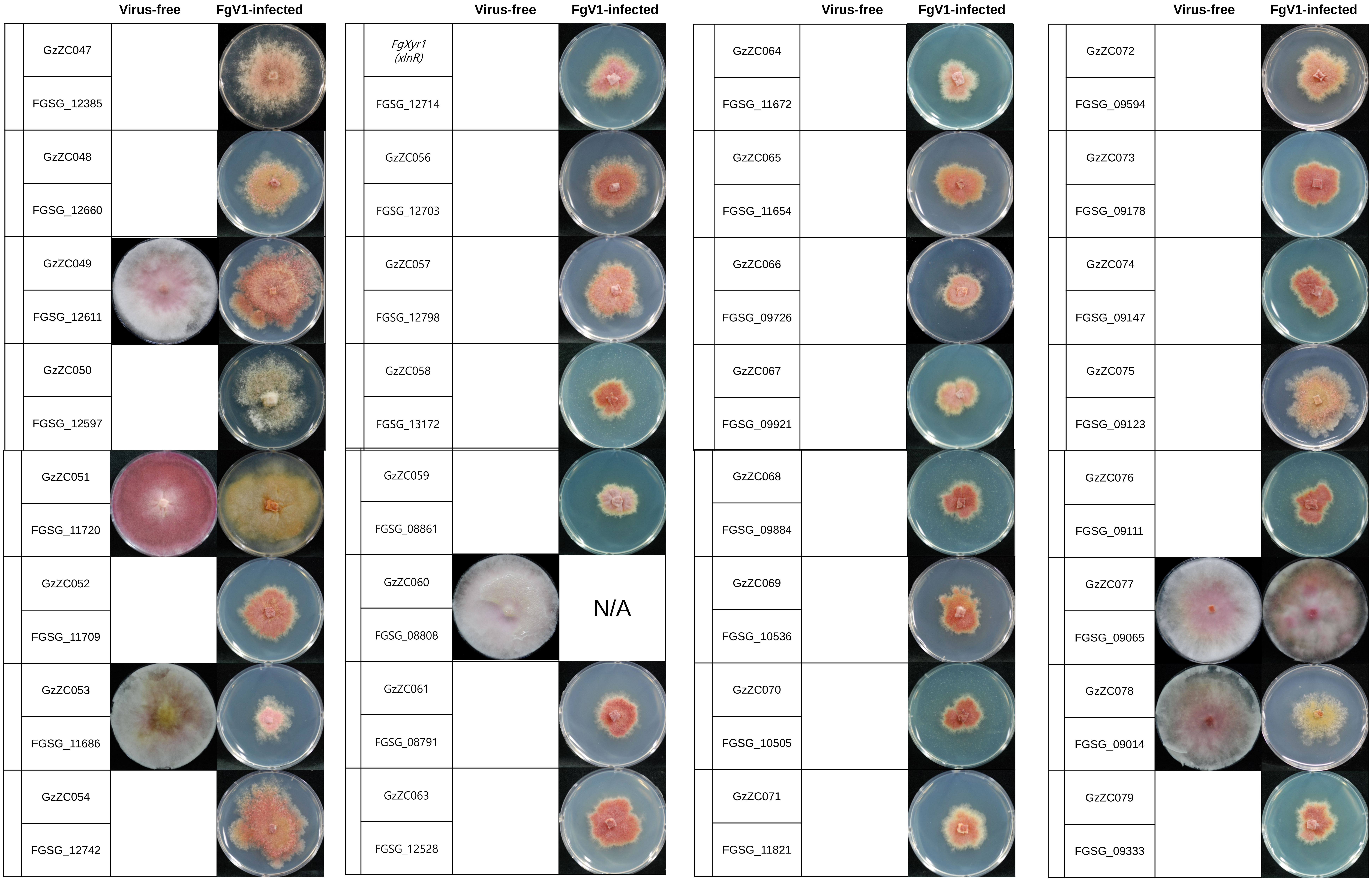

Virus-free
FgV1-infected
GzZC047
FGSG_12385
GzZC048
FGSG_12660
GzZC049
FGSG_12611
GzZC050
FGSG_12597
GzZC051
FGSG_11720
GzZC052
FGSG_11709
GzZC053
FGSG_11686
GzZC054
FGSG_12742
Virus-free
FgV1-infected
FgXyr1
(xlnR)
FGSG_12714
GzZC056
FGSG_12703
GzZC057
FGSG_12798
GzZC058
FGSG_13172
GzZC059
FGSG_08861
GzZC060
FGSG_08808
N/A
GzZC061
FGSG_08791
GzZC063
FGSG_12528
Virus-free
FgV1-infected
GzZC064
FGSG_11672
GzZC065
FGSG_11654
GzZC066
FGSG_09726
GzZC067
FGSG_09921
GzZC068
FGSG_09884
GzZC069
FGSG_10536
GzZC070
FGSG_10505
GzZC071
FGSG_11821
Virus-free
FgV1-infected
GzZC072
FGSG_09594
GzZC073
FGSG_09178
GzZC074
FGSG_09147
GzZC075
FGSG_09123
GzZC076
FGSG_09111
GzZC077
FGSG_09065
GzZC078
FGSG_09014
GzZC079
FGSG_09333

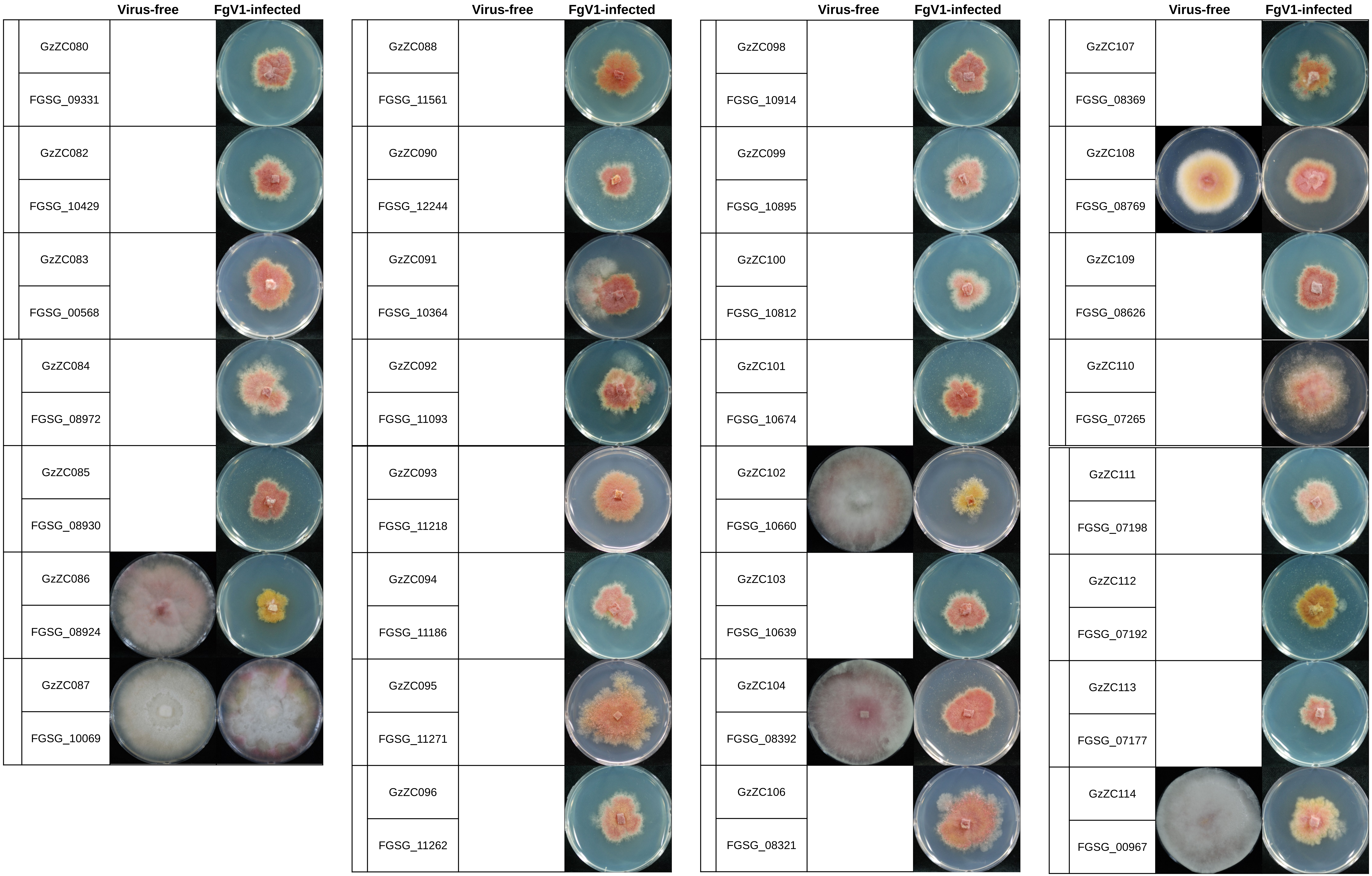

Virus-free
FgV1-infected
GzZC080
FGSG_09331
GzZC082
FGSG_10429
GzZC083
FGSG_00568
GzZC084
FGSG_08972
GzZC085
FGSG_08930
GzZC086
FGSG_08924
GzZC087
FGSG_10069
Virus-free
FgV1-infected
GzZC088
FGSG_11561
GzZC090
FGSG_12244
GzZC091
FGSG_10364
GzZC092
FGSG_11093
GzZC093
FGSG_11218
GzZC094
FGSG_11186
GzZC095
FGSG_11271
GzZC096
FGSG_11262
Virus-free
FgV1-infected
GzZC098
FGSG_10914
GzZC099
FGSG_10895
GzZC100
FGSG_10812
GzZC101
FGSG_10674
GzZC102
FGSG_10660
GzZC103
FGSG_10639
GzZC104
FGSG_08392
GzZC106
FGSG_08321
Virus-free
FgV1-infected
GzZC107
FGSG_08369
GzZC108
FGSG_08769
GzZC109
FGSG_08626
GzZC110
FGSG_07265
GzZC111
FGSG_07198
GzZC112
FGSG_07192
GzZC113
FGSG_07177
GzZC114
FGSG_00967

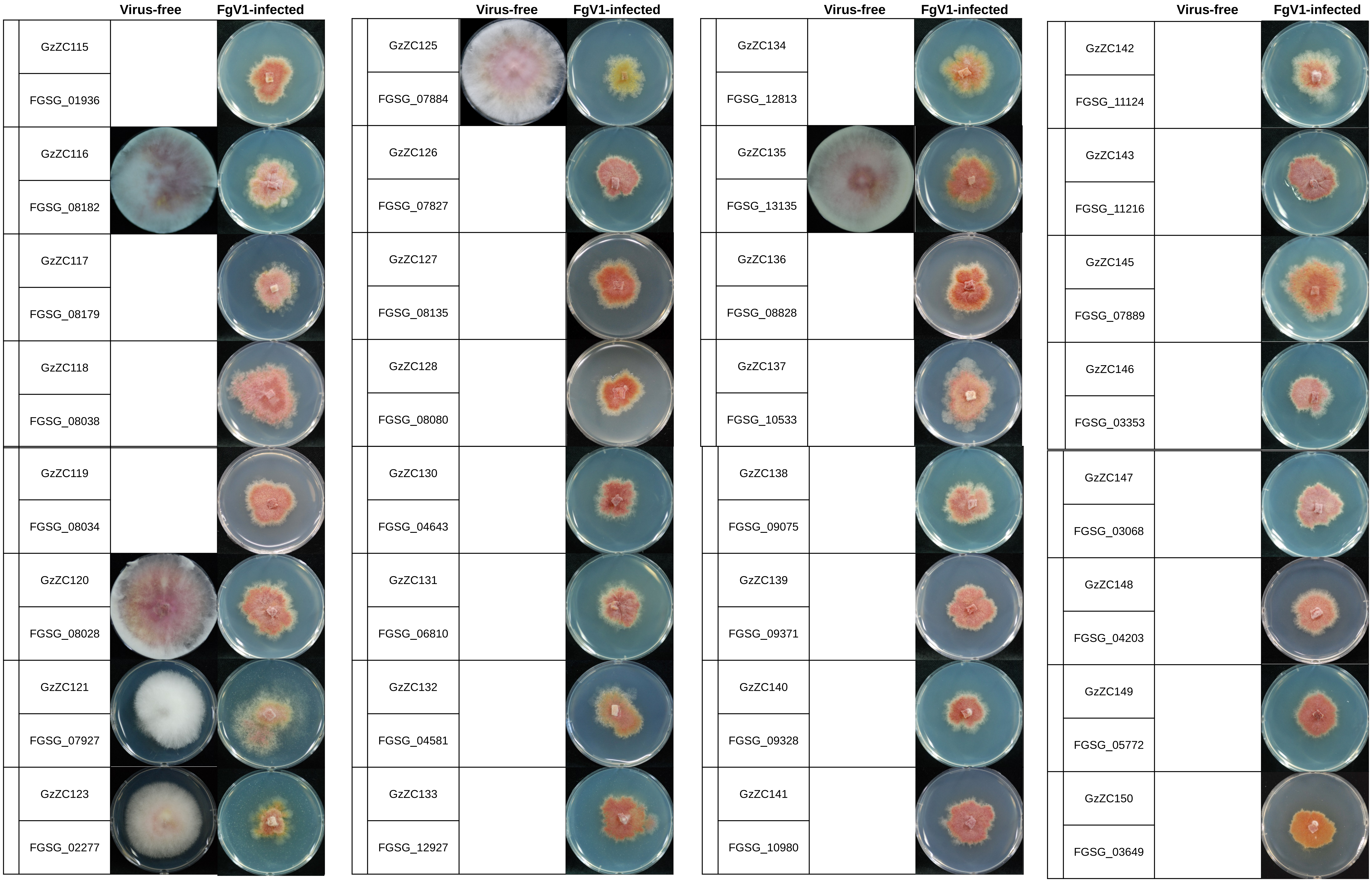
Virus-free
FgV1-infected
GzZC115
FGSG_01936
GzZC116
FGSG_08182
GzZC117
FGSG_08179
GzZC118
FGSG_08038
GzZC119
FGSG_08034
GzZC120
FGSG_08028
GzZC121
FGSG_07927
GzZC123
FGSG_02277
Virus-free
FgV1-infected
GzZC125
FGSG_07884
GzZC126
FGSG_07827
GzZC127
FGSG_08135
GzZC128
FGSG_08080
GzZC130
FGSG_04643
GzZC131
FGSG_06810
GzZC132
FGSG_04581
GzZC133
FGSG_12927
Virus-free
FgV1-infected
GzZC134
FGSG_12813
GzZC135
FGSG_13135
GzZC136
FGSG_08828
GzZC137
FGSG_10533
GzZC138
FGSG_09075
GzZC139
FGSG_09371
GzZC140
FGSG_09328
GzZC141
FGSG_10980
Virus-free
FgV1-infected
GzZC142
FGSG_11124
GzZC143
FGSG_11216
GzZC145
FGSG_07889
GzZC146
FGSG_03353
GzZC147
FGSG_03068
GzZC148
FGSG_04203
GzZC149
FGSG_05772
GzZC150
FGSG_03649

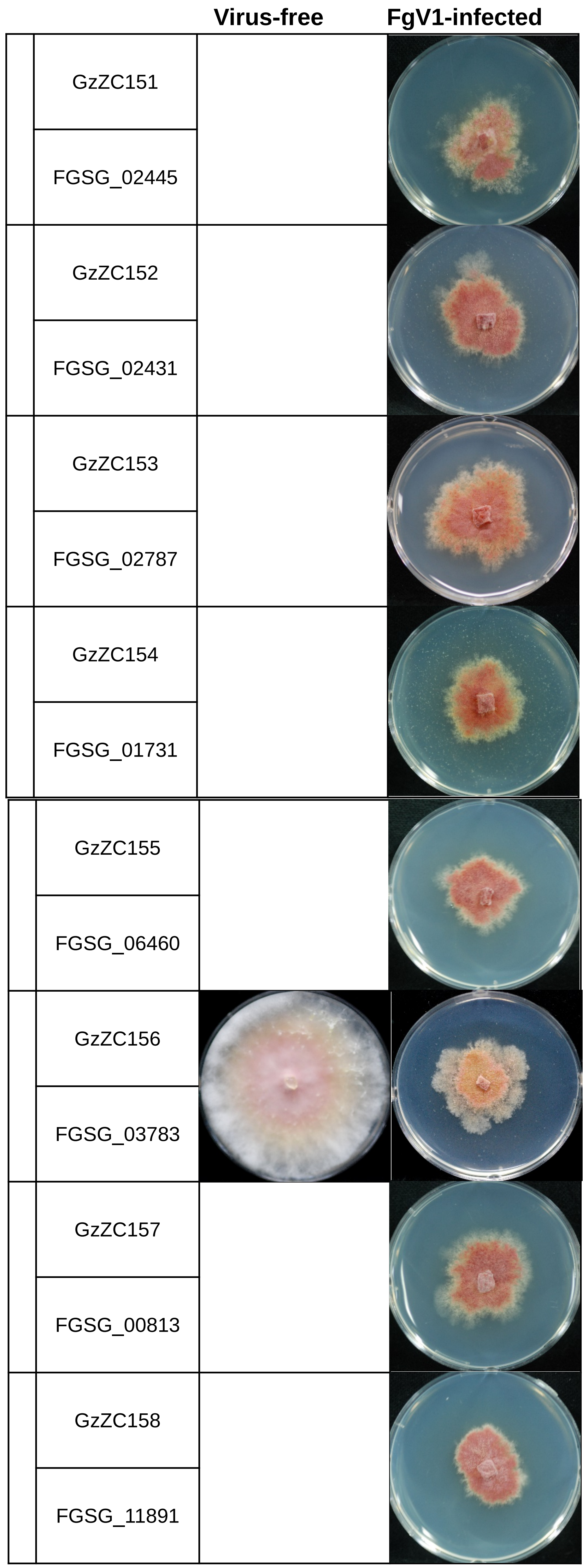
Virus-free
FgV1-infected
GzZC151
FGSG_02445
GzZC152
FGSG_02431
GzZC153
FGSG_02787
GzZC154
FGSG_01731
GzZC155
FGSG_06460
GzZC156
FGSG_03783
GzZC157
FGSG_00813
GzZC158
FGSG_11891
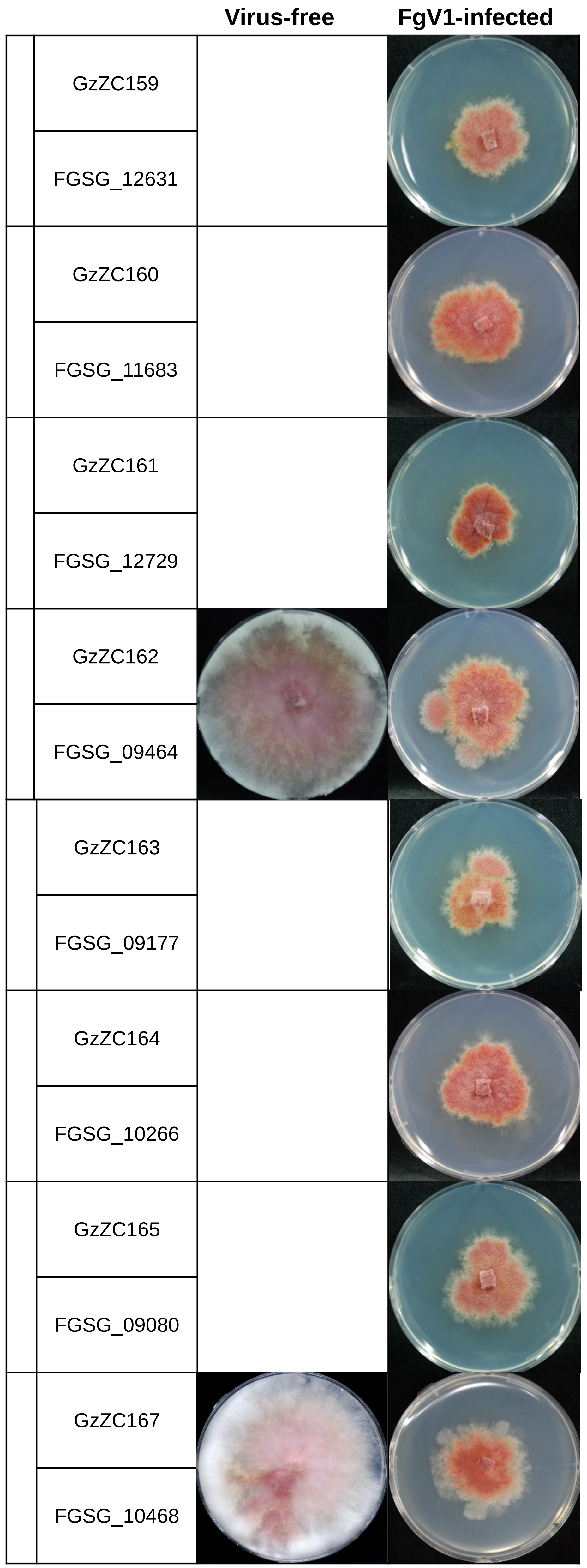
Virus-free
FgV1-infected
GzZC159
FGSG_12631
GzZC160
FGSG_11683
GzZC161
FGSG_12729
GzZC162
FGSG_09464
GzZC163
FGSG_09177
GzZC164
FGSG_10266
GzZC165
FGSG_09080
GzZC167
FGSG_10468
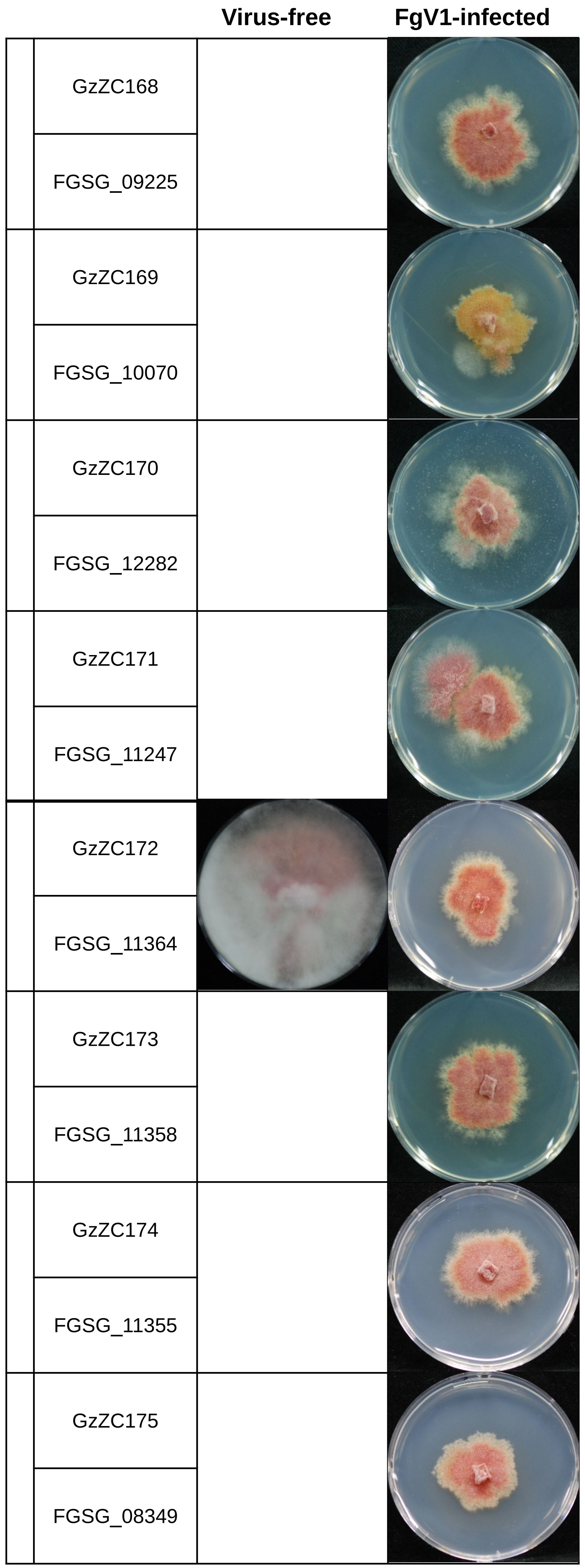
Virus-free
FgV1-infected
GzZC168
FGSG_09225
GzZC169
FGSG_10070
GzZC170
FGSG_12282
GzZC171
FGSG_11247
GzZC172
FGSG_11364
GzZC173
FGSG_11358
GzZC174
FGSG_11355
GzZC175
FGSG_08349
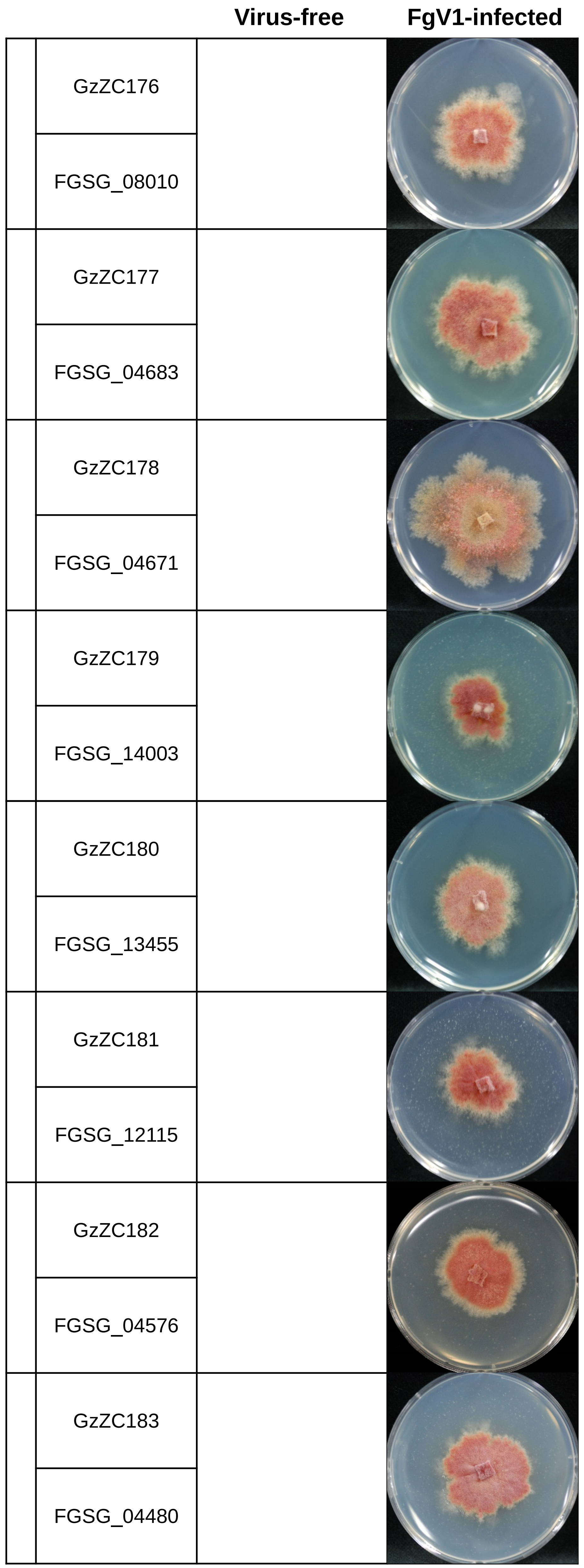
Virus-free
FgV1-infected
GzZC176
FGSG_08010
GzZC177
FGSG_04683
GzZC178
FGSG_04671
GzZC179
FGSG_14003
GzZC180
FGSG_13455
GzZC181
FGSG_12115
GzZC182
FGSG_04576
GzZC183
FGSG_04480

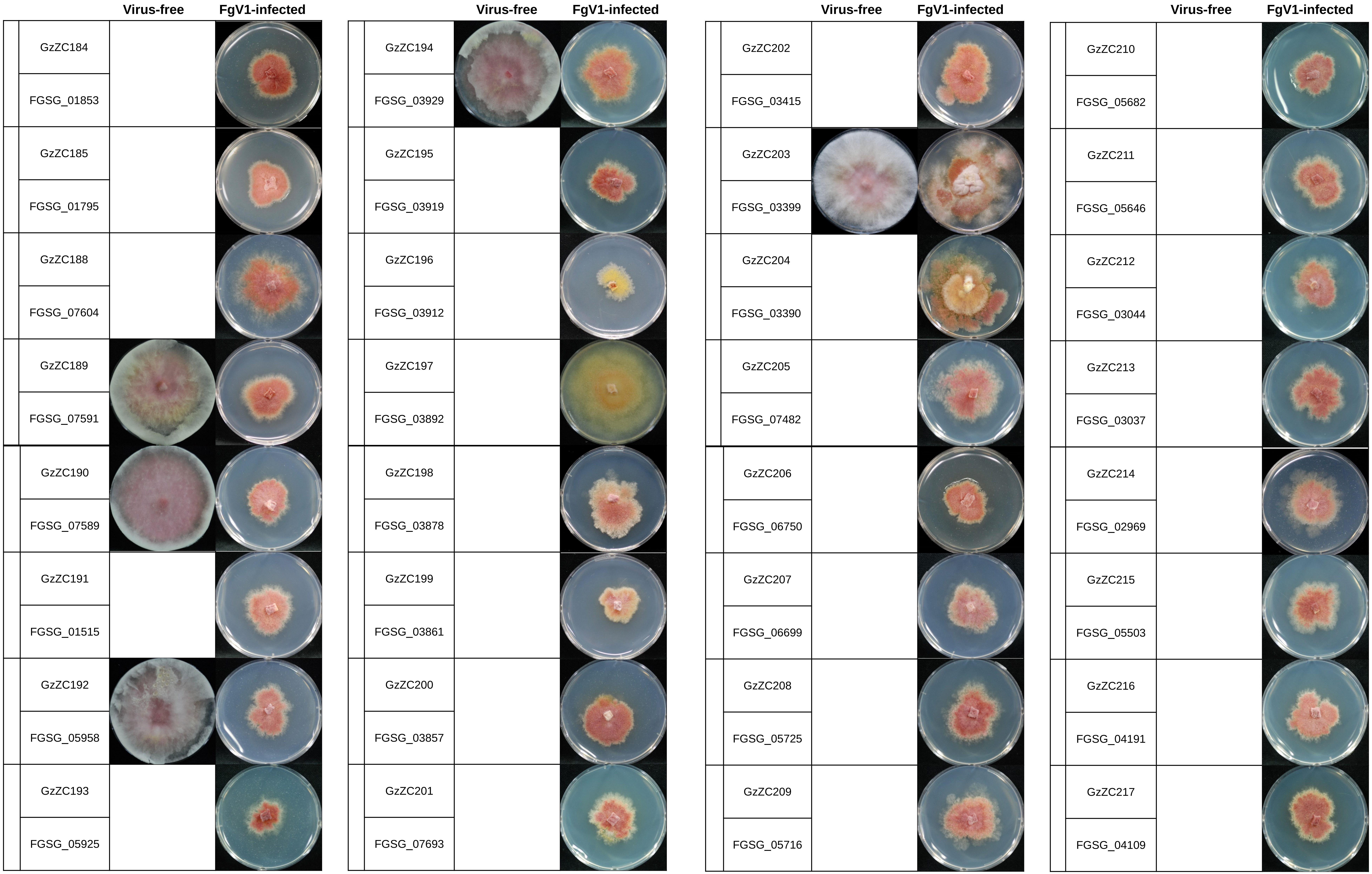
Virus-free
FgV1-infected
GzZC184
FGSG_01853
GzZC185
FGSG_01795
GzZC188
FGSG_07604
GzZC189
FGSG_07591
GzZC190
FGSG_07589
GzZC191
FGSG_01515
GzZC192
FGSG_05958
GzZC193
FGSG_05925
Virus-free
FgV1-infected
GzZC194
FGSG_03929
GzZC195
FGSG_03919
GzZC196
FGSG_03912
GzZC197
FGSG_03892
GzZC198
FGSG_03878
GzZC199
FGSG_03861
GzZC200
FGSG_03857
GzZC201
FGSG_07693
Virus-free
FgV1-infected
GzZC202
FGSG_03415
GzZC203
FGSG_03399
GzZC204
FGSG_03390
GzZC205
FGSG_07482
GzZC206
FGSG_06750
GzZC207
FGSG_06699
GzZC208
FGSG_05725
GzZC209
FGSG_05716
Virus-free
FgV1-infected
GzZC210
FGSG_05682
GzZC211
FGSG_05646
GzZC212
FGSG_03044
GzZC213
FGSG_03037
GzZC214
FGSG_02969
GzZC215
FGSG_05503
GzZC216
FGSG_04191
GzZC217
FGSG_04109

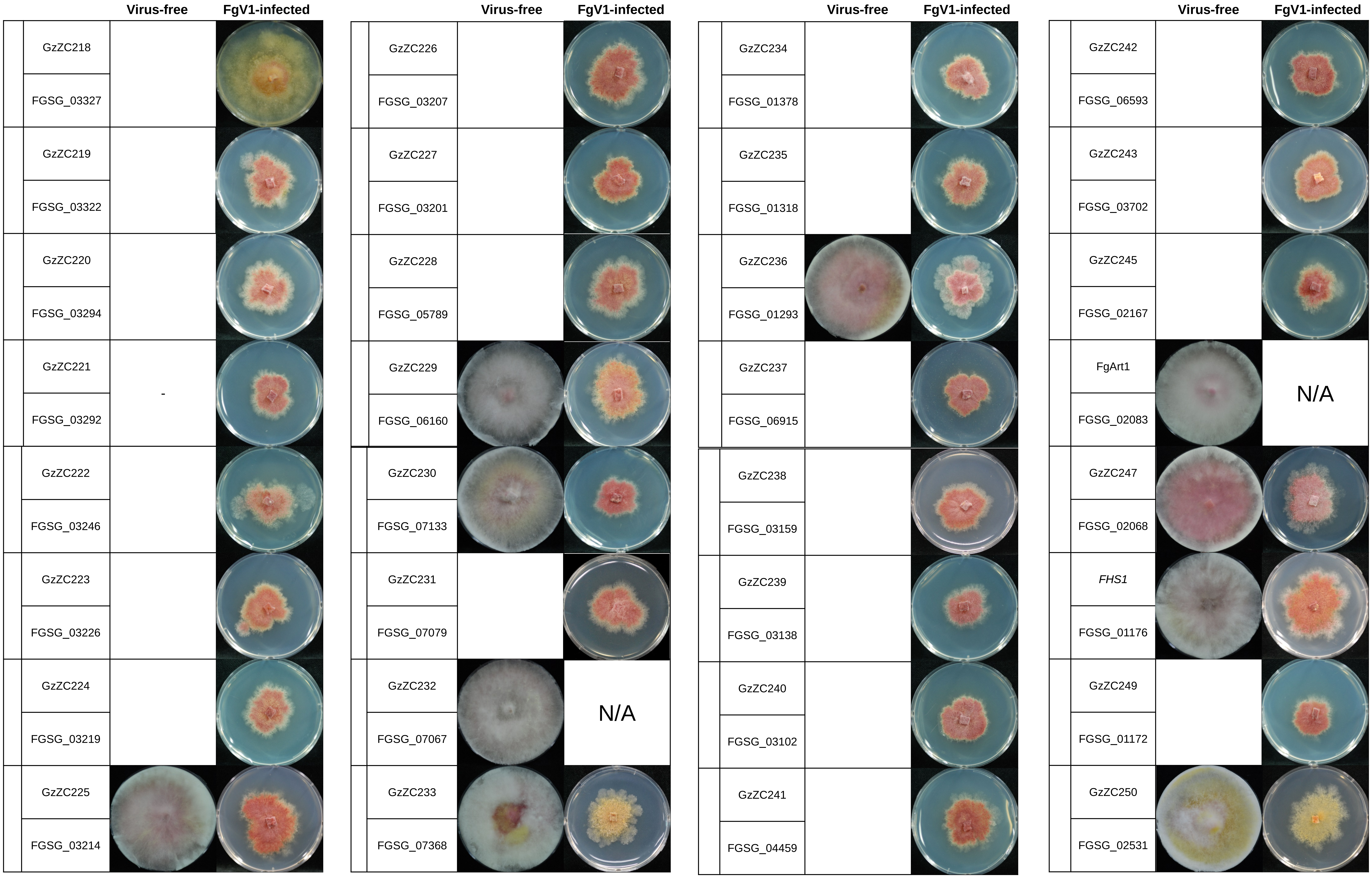
Virus-free
FgV1-infected
GzZC218
FGSG_03327
GzZC219
FGSG_03322
GzZC220
FGSG_03294
GzZC221
FGSG_03292
-
GzZC222
FGSG_03246
GzZC223
FGSG_03226
GzZC224
FGSG_03219
GzZC225
FGSG_03214
Virus-free
FgV1-infected
GzZC226
FGSG_03207
GzZC227
FGSG_03201
GzZC228
FGSG_05789
GzZC229
FGSG_06160
GzZC230
FGSG_07133
GzZC231
FGSG_07079
GzZC232
FGSG_07067
N/A
GzZC233
FGSG_07368
Virus-free
FgV1-infected
GzZC234
FGSG_01378
GzZC235
FGSG_01318
GzZC236
FGSG_01293
GzZC237
FGSG_06915
GzZC238
FGSG_03159
GzZC239
FGSG_03138
GzZC240
FGSG_03102
GzZC241
FGSG_04459
Virus-free
FgV1-infected
GzZC242
FGSG_06593
GzZC243
FGSG_03702
GzZC245
FGSG_02167
FgArt1
FGSG_02083
N/A
GzZC247
FGSG_02068
FHS1
FGSG_01176
GzZC249
FGSG_01172
GzZC250
FGSG_02531

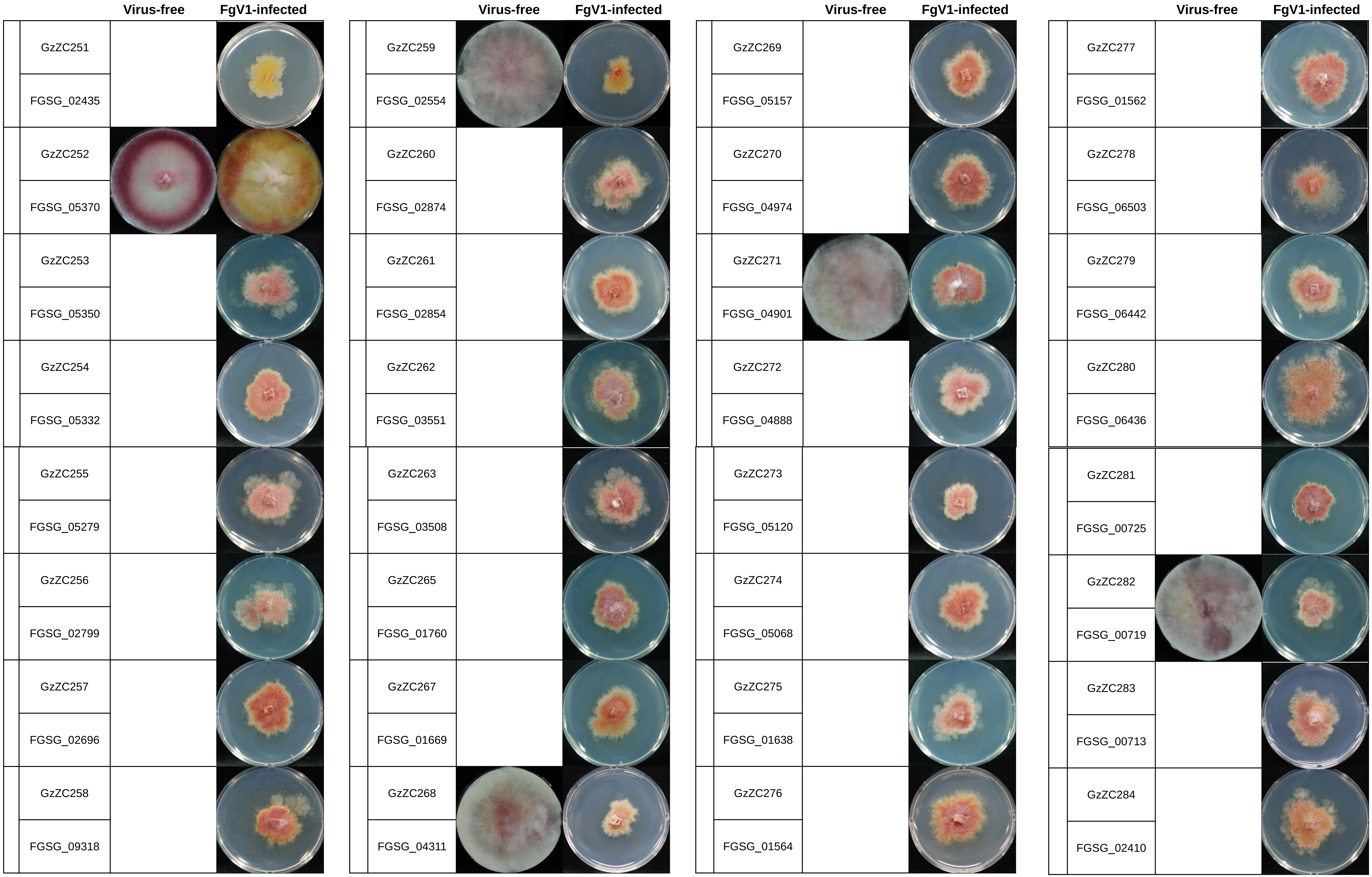
Virus-free
FgV1-infected
GzZC251
FGSG_02435
GzZC252
FGSG_05370
GzZC253
FGSG_05350
GzZC254
FGSG_05332
GzZC255
FGSG_05279
GzZC256
FGSG_02799
GzZC257
FGSG_02696
GzZC258
FGSG_09318
GzZC259
FGSG_02554
GzZC260
FGSG_02874
GzZC261
FGSG_02854
GzZC262
FGSG_03551
GzZC263
FGSG_03508
GzZC265
FGSG_01760
GzZC267
FGSG_01669
GzZC268
FGSG_04311
GzZC269
FGSG_05157
GzZC270
FGSG_04974
GzZC271
FGSG_04901
GzZC272
FGSG_04888
GzZC273
FGSG_05120
GzZC274
FGSG_05068
GzZC275
FGSG_01638
GzZC276
FGSG_01564
GzZC277
FGSG_01562
GzZC278
FGSG_06503
GzZC279
FGSG_06442
GzZC280
FGSG_06436
GzZC281
FGSG_00725
GzZC282
FGSG_00719
GzZC283
FGSG_00713
GzZC284
FGSG_02410

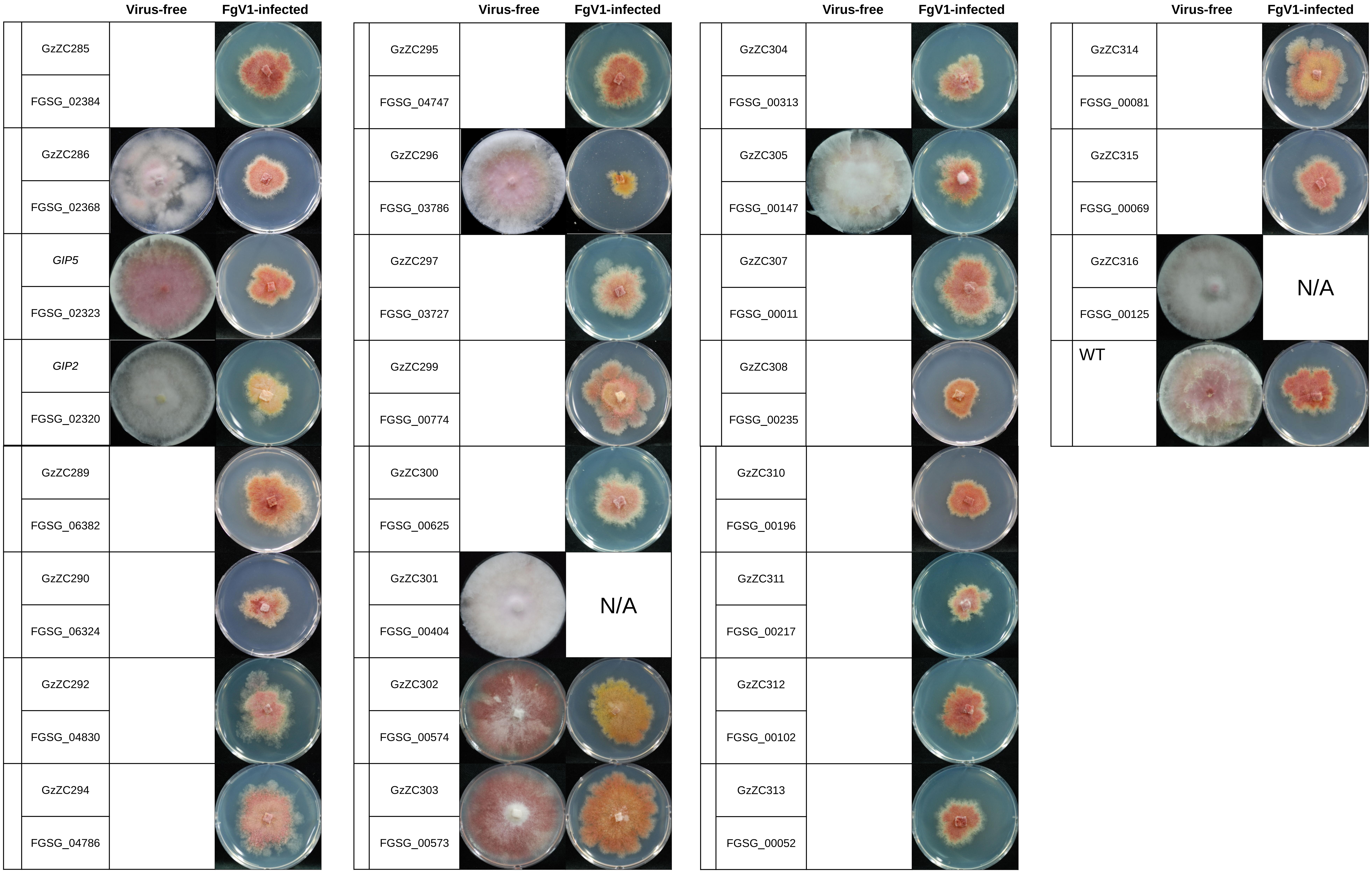

Virus-free
FgV1-infected
GzZC285
FGSG_02384
GzZC286
FGSG_02368
GIP5
FGSG_02323
GIP2
FGSG_02320
GzZC289
FGSG_06382
GzZC290
FGSG_06324
GzZC292
FGSG_04830
GzZC294
FGSG_04786
Virus-free
FgV1-infected
GzZC295
FGSG_04747
GzZC296
FGSG_03786
GzZC297
FGSG_03727
GzZC299
FGSG_00774
GzZC300
FGSG_00625
GzZC301
FGSG_00404
N/A
GzZC302
FGSG_00574
GzZC303
FGSG_00573
Virus-free
FgV1-infected
GzZC304
FGSG_00313
GzZC305
FGSG_00147
GzZC307
FGSG_00011
GzZC308
FGSG_00235
GzZC310
FGSG_00196
GzZC311
FGSG_00217
GzZC312
FGSG_00102
GzZC313
FGSG_00052
Virus-free
FgV1-infected
GzZC314
FGSG_00081
GzZC315
FGSG_00069
GzZC316
FGSG_00125
N/A
WT
