# Supplementary material for: A Phenome-Wide Association Study of the Effects of Fusarium graminearum Transcription Factors on Fusarium Graminearum Virus 1 Infection
Source: Front Microbiol. 2021 Feb 11;12:622261. doi: 10.3389/fmicb.2021.622261 (PMC7904688; doi:10.3389/fmicb.2021.622261)
Supplement: Supplementary file 4 [file Table_3.DOCX]

**Supplementary Table 3. Selected transcription factor deletion mutants that showed multiple defect phenotypes.**

| **Locus ID^a^** | **Gene name** | **FgV1 infection** | **Multiple defects in  deletion mutant^b^** |
| --- | --- | --- | --- |
| FGSG_04220 | *FgSwi6* | 2 | NP, Vir, DON, MM |
| FGSG_10129 | *FgStuA* | 3 | NP, Vir, DON, MM |
| FGSG_10384 | *GzAPSES004* | N/A | NP, Vir, DON, MM |
| FGSG_06071 | *GzAT001* | N/A | NP, Vir, MM |
| FGSG_06291 | *GzBrom002* | N/A | NP, Vir, DON, MM |
| FGSG_01555 | *ZIF1* | 2 | NP, Vir, DON |
| FGSG_05171 | *GzbZIP007* | 2 | NP, Vir, DON, MM |
| FGSG_00477 | *GzC2H003* | 1 | NP, Vir, MM |
| FGSG_01022 | *GzC2H007* | 2 | NP, Vir, DON |
| FGSG_01106 | *FgArs2* | 1 | Vir, DON |
| FGSG_01350 | *GzC2H014* | 2 | NP, Vir, DON, MM |
| FGSG_04134 | *GzCON7* | 2 | NP, Vir, DON, MM |
| FGSG_06871 | *FgMSN2* | 3 | NP, Vir, MM |
| FGSG_10517 | *GzC2H090* | 2 | NP, Vir, DON, MM |
| FGSG_13711 | *FgCrz1A* | 3 | NP, Vir, DON, MM |
| FGSG_13746 | *GzNot002* | 1 | NP, Vir, DON, MM |
| FGSG_05304 | *FCT2(FgHLTF1)* | 1 | NP, Vir, DON, MM |
| FGSG_00385 | *FgNHP6A* | 1 | NP, Vir, DON, MM |
| FGSG_06948 | *Gzscp* | 3 | NP, Vir, DON, MM |
| FGSG_09339 | *GzMADS003* | 3 | NP, Vir, DON |
| FGSG_00324 | *MYT3* | 1 | NP, Vir, DON, MM |
| FGSG_12781 | *GzMyb017* | 2 | NP, Vir, DON, MM |
| FGSG_09992 | *GzNH001* | N/A | NP, Vir, DON, MM |
| FGSG_08737 | *Hex1* | 2 | NP, Vir |
| FGSG_13120 | *GzOB047* | 2 | NP, Vir, DON, MM |
| FGSG_01665 | *FgFSR1* | N/A | NP, Vir, DON, MM |
| FGSG_06228 | *FgFlbA* | 2 | NP, Vir, DON |
| FGSG_08572 | *GzWing019* | 3 | NP, Vir, DON, MM |
| FGSG_08719 | *GzWing020* | 2 | NP, Vir, DON, MM |
| FGSG_10716 | *GzCCHC011* | 2 | NP, Vir, DON, MM |
| FGSG_06542 | *GzDHHC003* | 2 | NP |
| FGSG_10069 | *GzZC087* | 3 | NP, Vir, MM |
| FGSG_08769 | *GzZC108* | 3 | NP, Vir, DON, MM |
| FGSG_07067 | *GzZC232* | N/A | NP, Vir, MM |
| FGSG_00574 | *GzZC302* | 3 | NP, Vir, MM |

^a^Colored in gene locus line represents TF deletion mutants that were not related with stress response.

^b^Analysis data were extracted and modified from the original report (Son et al., 2011). List of TF mutants showing multiple defects. NP, no perithecia development; Vir, reduced virulence; DON, abnormal deoxynivaleol production; MM, growth defect in mycelial growth on minimal medium.

**Supplementary Table 4. Expression level of putative TF genes following FgV1 infection.**

| **Locus ID** | **Gene name** | **qRT-PCR^a^** | | **Note** |
| --- | --- | --- | --- | --- |
|  |  | **WT-VF** | **WT-VI** |  |
| FGSG_04220 | *FgSwi6* | 1.06±0.21 | 0.37±0.21* | Gene deletion phenotype (virus-free) showed similar to WT-VI. |
| FGSG_13746 | *GzNot002* | 1.20±0.24 | 0.89±0.19 |  |
| FGSG_10517 | *GzC2H090* | 0.83±0.23 | 0.33±0.23 |  |
| FGSG_08572 | *GzWing019* | 1.00±0.16 | 0.65±0.12 |  |
| FGSG_13711 | *FgCrz1A* | 1.16±0.38 | 0.62±0.23 |  |
| FGSG_12264 | *GzRad003* | 0.96±0.29 | 0.43±0.10* | Virus transmission was not possible to those TF gene deletion mutant group. |
| FGSG_06380 | *GzZC030* | 0.87±0.25 | 0.41±0.16* |  |
| FGSG_12094 | *GzZC044* | 0.98±0.09 | 0.34±0.16* |  |
| FGSG_08808 | *GzZC060* | 1.01±0.12 | 0.62±0.19 |  |
| FGSG_02083 | *FgArt1* | 0.90±0.34 | 0.37±0.10* |  |
| FGSG_00404 | *GzZC301* | 0.90±0.14 | 1.31±0.23** |  |
| FGSG_00125 | *GzZC316* | 1.23±0.23 | 2.67±0.72** |  |
| FGSG_00420 | *GzFET5* | 0.82±0.32 | 0.43±0.11 |  |
| FGSG_02150 | *GzZC026* | 1.02±0.10 | 0.24±0.13* |  |
| FGSG_08865 | *GzOB035* | 0.94±0.15 | 2.08±0.57** | FgV1-infected TF deletion mutant belong to the Group 1 |
| FGSG_13828 | *GzZC040* | 0.88±0.17 | 3.27±0.97** |  |
| FGSG_08924 | *GzZC086* | 0.91±0.25 | 0.97±0.19 |  |
| FGSG_01106 | *FgArs2* | 0.85±0.15 | 0.44±0.09 |  |
| FGSG_05304 | *FCT2(FgHLTF1)* | 0.96±0.16 | 0.64±0.13 |  |
| FGSG_12345 | *GzbZIP019* | 0.89±0.46 | 0.74±0.37 | FgV1-infected TF deletion mutant belong to the Group 3 |
| FGSG_09339 | *GzMADS003* | 0.98±0.19 | 0.37±0.17* |  |
| FGSG_08455 | *GzMIZ001* | 0.98±0.20 | 0.20±0.07* |  |
| FGSG_03873 | *GzZC021* | 0.80±0.18 | 0.17±0.10* |  |
| FGSG_13625 | *GzZC034* | 0.42±0.27 | 0.19±0.09 |  |
| FGSG_12742 | *GzZC054* | 0.83±0.10 | 1.72±0.32** |  |

^a^Quantification of target gene expression using real-time RT-PCR. cDNAs were generated from total RNA samples obtained after 120 h of incubation. *EF1α* and *UBH* gene transcripts were used as internal controls. Values are means (+ SD) of two biological replicates with at least two experimental replications.

*Asterisk indicates data are significantly differed (P < 0.05) from the mean for virus-free GZ03639 wild-type strain based on LSD test.

**Supplementary Table 5. Validation of putative TF gene expression levels using qRT-PCR.**

| **Locus ID**^a^ | **Gene name** | **RNA-Seq  (p<0.05)**^b^ | **qRT-PCR^c^** | | **ΔTF**^d^ |
| --- | --- | --- | --- | --- | --- |
|  |  |  | **WT-VF** | **WT-VI** |  |
| FGSG_09286 | *GzbZIP015* | 4.13 | 1.00±0.14 | 1.17±0.22 | G2 |
| FGSG_11799 | *GzC2H095* | 3.54 | 1.05±0.14 | 0.39±0.15* | G2 |
| FGSG_00217 | *GzZC311* | 2.89 | 0.98±0.13 | 2.93±1.43* | G1 |
| FGSG_05370 | *GzZC252* | 3.59 | 0.99±0.25 | 3.52±2.00* | G3 |
| FGSG_07052 | *GzC2H047* | -3.02 | 0.78±0.20 | 0.50±0.18 | G2 |
| FGSG_07735 | *GzC2H053* | -3.49 | 0.99±0.16 | 0.24±0.10* | G2 |
| FGSG_06110 | *GzOB021* | -3.99 | 0.97±0.16 | 0.37±0.15* | G2 |
| FGSG_02130 | *GzNF001* | -4.56 | 0.86±0.10 | 0.23±0.08* | G2 |

^a^Among 23 putative TF genes that were differentially expressed following FgV1 infection (log_2_ fold changes > 2, p-value < 0.05), eight genes were selected for validation of RNA-Seq data.

^b^Relative expression ratio of putative TF genes in FgV1-infected *F. graminearum* PH-1 compared to the virus-free *F. graminearum* PH-1 at 120 h post-inoculation. Analysis data for differentially expressed genes were extracted and from the original report (Lee et al.,2014).

^c^Quantification of the target gene expression using real-time RT-PCR. cDNAs were generated from total RNA samples obtained after 120 h of incubation. *EF1α* and *UBH* gene transcripts were used as internal controls. Values are means (+ SD) of two biological replicates with at least two experimental replications.

*Asterisk indicates data are significantly differed (P < 0.05) from the mean for virus-free GZ03639 wild-type strain based on LSD test.

^d^Designated group of each TF deletion mutant according to mycelial growth.
